# Wearable aptamer-field-effect transistor sensing system for noninvasive cortisol monitoring

**DOI:** 10.1126/sciadv.abk0967

**Published:** 2022-01-05

**Authors:** Bo Wang, Chuanzhen Zhao, Zhaoqing Wang, Kyung-Ae Yang, Xuanbing Cheng, Wenfei Liu, Wenzhuo Yu, Shuyu Lin, Yichao Zhao, Kevin M. Cheung, Haisong Lin, Hannaneh Hojaiji, Paul S. Weiss, Milan N. Stojanović, A. Janet Tomiyama, Anne M. Andrews, Sam Emaminejad

**Affiliations:** 1 Interconnected and Integrated Bioelectronics Lab (I^2^BL), Department of Electrical and Computer Engineering, University of California, Los Angeles, Los Angeles, CA 90095, USA.; 2 Department of Chemistry and Biochemistry, University of California, Los Angeles, Los Angeles, CA 90095, USA.; 3 California NanoSystems Institute, University of California, Los Angeles, Los Angeles, CA 90095, USA.; 4 Division of Experimental Therapeutics, Department of Medicine, Columbia University, New York, NY 10032, USA.; 5 Department of Materials Science and Engineering, University of California, Los Angeles, Los Angeles, CA 90095, USA.; 6 Department of Bioengineering, University of California, Los Angeles, Los Angeles, CA 90095, USA.; 7 Department of Biomedical Engineering, Columbia University, New York, NY 10032, USA.; 8 Department of Psychology, University of California, Los Angeles, Los Angeles, CA 90095, USA.; 9 Department of Psychiatry and Biobehavioral Sciences, University of California, Los Angeles, Los Angeles, CA 90095, USA.; 10 Semel Institute for Neuroscience and Human Behavior, University of California, Los Angeles, Los Angeles, CA 90095, USA.; 11 Hatos Center for Neuropharmacology, University of California, Los Angeles, Los Angeles, CA 90095, USA.

## Abstract

Wearable technologies for personalized monitoring require sensors that track biomarkers often present at low levels. Cortisol—a key stress biomarker—is present in sweat at low nanomolar concentrations. Previous wearable sensing systems are limited to analytes in the micromolar-millimolar ranges. To overcome this and other limitations, we developed a flexible field-effect transistor (FET) biosensor array that exploits a previously unreported cortisol aptamer coupled to nanometer-thin-film In_2_O_3_ FETs. Cortisol levels were determined via molecular recognition by aptamers where binding was transduced to electrical signals on FETs. The physiological relevance of cortisol as a stress biomarker was demonstrated by tracking salivary cortisol levels in participants in a Trier Social Stress Test and establishing correlations between cortisol in diurnal saliva and sweat samples. These correlations motivated the development and on-body validation of an aptamer-FET array–based smartwatch equipped with a custom, multichannel, self-referencing, and autonomous source measurement unit enabling seamless, real-time cortisol sweat sensing.

## Teaser

A smartwatch was developed for noninvasive stress biomarker data acquisition that provides real-time feedback to the wearer.

## INTRODUCTION

Wearable monitoring technologies have the power to transform health care by providing personalized, actionable feedback enabling changes in physical and cognitive performance and the adoption of more healthier lifestyle routines. Wearable sensors that detect and quantify biomarkers in retrievable biofluids provide specific information on human dynamic physiological and psychological status (*1*, *2*). On-body sensing systems have been used to make measurements of physiologically informative indices in sweat, including pH and electrolyte, metabolite, or nutrient levels (*3*–*6*).

Nevertheless, many low concentration, potentially informative biomarkers are not accessible by wearable sensing systems. Included are hormones and other biomarkers present at (sub)nanomolar levels in the presence of high-concentration interferants in native biofluids (*1*). Shortcomings are inherent at the sensor and systems levels. Hence, the potential utility of wearable sensors remains limited to a small number of narrow applications (*1*). Moreover, existing wearable systems have neither the resolution nor dynamic capabilities needed to capture physiologically relevant changes in biomarker levels accurately and seamlessly.

**Fig. 1.**
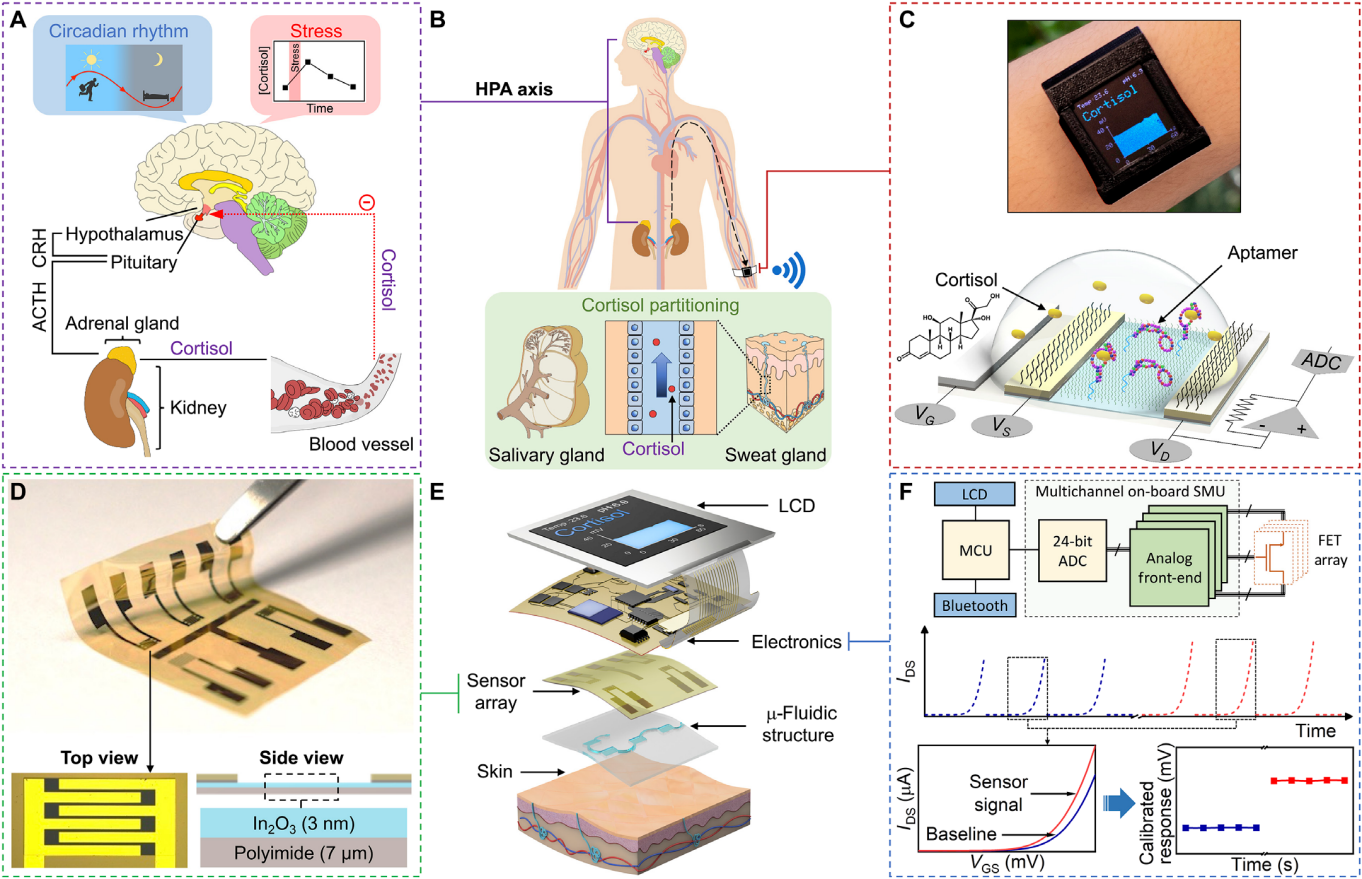
Noninvasive cortisol biomarker monitoring using a wearable aptamer-field-effect transistor sensing system. (**A**) The hypothalamus-pituitary-adrenal (HPA) axis controls cortisol levels in response to circadian rhythm and stress. ACTH, adrenocorticotropic hormone; CRH, corticotropin-releasing hormone. (**B**) The fraction of circulating cortisol not bound to blood plasma proteins is available for excretion by salivary and sweat glands. (**C**) Saliva and sweat samples can be analyzed by an aptamer-field-effect transistor (FET) sensing system. Top: Photograph of an aptamer-FET-enabled biosensing smartwatch. Bottom: Schematic illustration of cortisol sensing by an aptamer-FET sensor. *V*_G_, gate voltage; *V*_S_, source voltage; *V*_D_, drain voltage; ADC, analog-digital converter. (**D**) Photograph of a FET sensor array with In_2_O_3_ semiconductor channels fabricated on a flexible polyimide substrate. Schematic layers not to scale. (**E**) Expanded view of the key components of an aptamer-FET biosensing smartwatch. Liquid crystal display (LCD). (**F**) Overview of FET-array signal acquisition via a multichannel on-board source measurement unit (SMU). Data processing is via a microcontroller unit (MCU), display, and transmission. *I*_DS_, source-drain current; *V*_GS_, gate voltage. Photo credit: Zhaoqing Wang, Yichao Zhao, UCLA.

Cortisol is a low-concentration biomarker that provides information on psychobiological states that is currently challenging for noninvasive monitoring. It is a key component of the stress-responsive hypothalamus-pituitary-adrenal axis (Fig. 1, A and B) (*7*). Cortisol dysregulation occurs in major depressive disorder, anxiety disorders, posttraumatic stress disorder, obesity, and Cushing’s and Addison’s diseases (*8*–*11*). Landmark studies have linked individual cortisol levels to neurobehavioral developmental trajectories and personal and team performance outcomes (*12*, *13*). Clinical studies have demonstrated significant correlations between free cortisol levels in saliva and blood (*1*, *14*, *15*). These associations are attributed to the relatively small size of cortisol (molecular weight, 362.5 g/mol) and its lipophilicity, which enable diffusion through glandular and capillary epithelial cell membranes. Similar correlations are hypothesized for cortisol in sweat due to comparable diffusive transport mechanisms from blood to sweat (Fig. 1B) (*1*, *16*).

Recent advances in biosensor development illustrate the importance and promise for noninvasive cortisol monitoring (*17*–*21*). Nonetheless, a wearable device for cortisol sensing using label-free and direct signal transduction, high sensitivity and selectivity, and real sample analysis capabilities (i.e., integration with electronics such that the sensor readout is processed autonomously and communicated wirelessly) has not yet been demonstrated (see table S1 for a comparative analysis of results from recent publications). For example, antibody-based cortisol sensors typically require the addition of external reagents and multistep manual operations constraining applications to ex situ settings (*4*, *18*, *19*, *22*–*24*), while molecularly imprinted polymer–based sensors can require the addition of redox probes for signal enhancement (*21*).

Here, to monitor low-concentration, small-molecule biomarkers, such as cortisol, in a wearable format, we designed, developed, and investigated a field-effect transistor (FET) array–based sensing system (Fig. 1C). This array exploits a newly identified cortisol aptamer (as a biorecognition element) coupled to the nanometer-thin In_2_O_3_ channels of FETs (as a signal transduction platform). Aptamer-based sensors show robust and selective target detection in minimally or undiluted biological samples, including blood, serum, and brain tissue (*25*–*28*). We have previously reported on the use of aptamer-FETs for highly sensitive and selective detection of small-molecule targets (e.g., glucose, serotonin, dopamine, and phenylalanine) in biofluids (*27*–*30*). Aptamer-FET detection of serotonin was stable after exposure to brain tissue (*28*, *31*). Target-induced conformational rearrangements of negatively charged aptamer phosphodiester backbones produce FET surface charge perturbations and, consequently, measurable electronic signals. The aptamer-based biorecognition process relies on the formation of aptamer-target complexes, which is independent of the chemical reactivity or intrinsic charge of the target molecules (*28*).

We fabricated aptamer-FETs on flexible polyimide substrates for wearable sensing applications (Fig. 1D) (*32*). Substrates were embedded in a tape-based thin-film microfluidic device to form a skin-adherable biofluid sampling, routing, and analysis module (Fig. 1E). The potential utility of using cortisol-aptamer-FET sensors to detect stress was determined by tracking salivary cortisol levels in participants in a Trier Social Stress Test (TSST) and then establishing correlations between cortisol in diurnal sweat and saliva samples.

Biologically relevant stress-associated increases in sweat cortisol levels motivated the development and on-body validation of an aptamer-FET array–based smartwatch. The wearable smartwatch was equipped with a custom on-board multichannel source measurement unit (SMU). The SMU featured continuous, high-resolution FET transfer curve acquisition capabilities (Fig. 1F). Readouts were processed using a normalization method to mitigate device-to-device variation (*33*).

Our approach overcomes critical shortcomings of previously reported transistor-based biosensors lacking system integration (*17*, *34*, *35*), which limit translation to wearable applications. By deploying an aptamer-FET array–based smartwatch, we achieve seamless and real-time biomarker data acquisition. Aptamer-FET sensors are generalizable and modular. They can be straightforwardly adapted in wearable and mobile formats for additional physiological biomarkers, including targets at low concentrations in sweat (or other body fluids) for which there are currently no available portable measurement technologies to advance personalized precision medicine.

## RESULTS

### Fabrication and characterization of flexible FETs

We have shown that quasi–two-dimensional (2D) In_2_O_3_ FETs fabricated on hard and soft substrates transduce surface interactions between tethered aptamers and their targets (*27*–*30*, *32*, *36*). Large semiconductor surface-to-volume ratios enable highly efficient signal transduction between aptamer-target binding events and semiconductor electric field perturbations (e.g., charge modulation). Moreover, aptamer-FETs are sensitive to targets having little or no charge under the high ionic strength conditions typically found in body fluids (*28*).

To fabricate FETs on flexible substrates for conformal skin contact, thin-film In_2_O_3_ was formed on polyimide via spin coating the In_2_O_3_ precursor followed by solution-processed sol-gel chemistry (*37*, *38*). The In_2_O_3_ layer was then patterned by photolithography and reactive ion etching to form the channel regions (fig. S1). Interdigitated Au/Ti electrodes were patterned to form source and drain contacts.

**Fig. 2.**
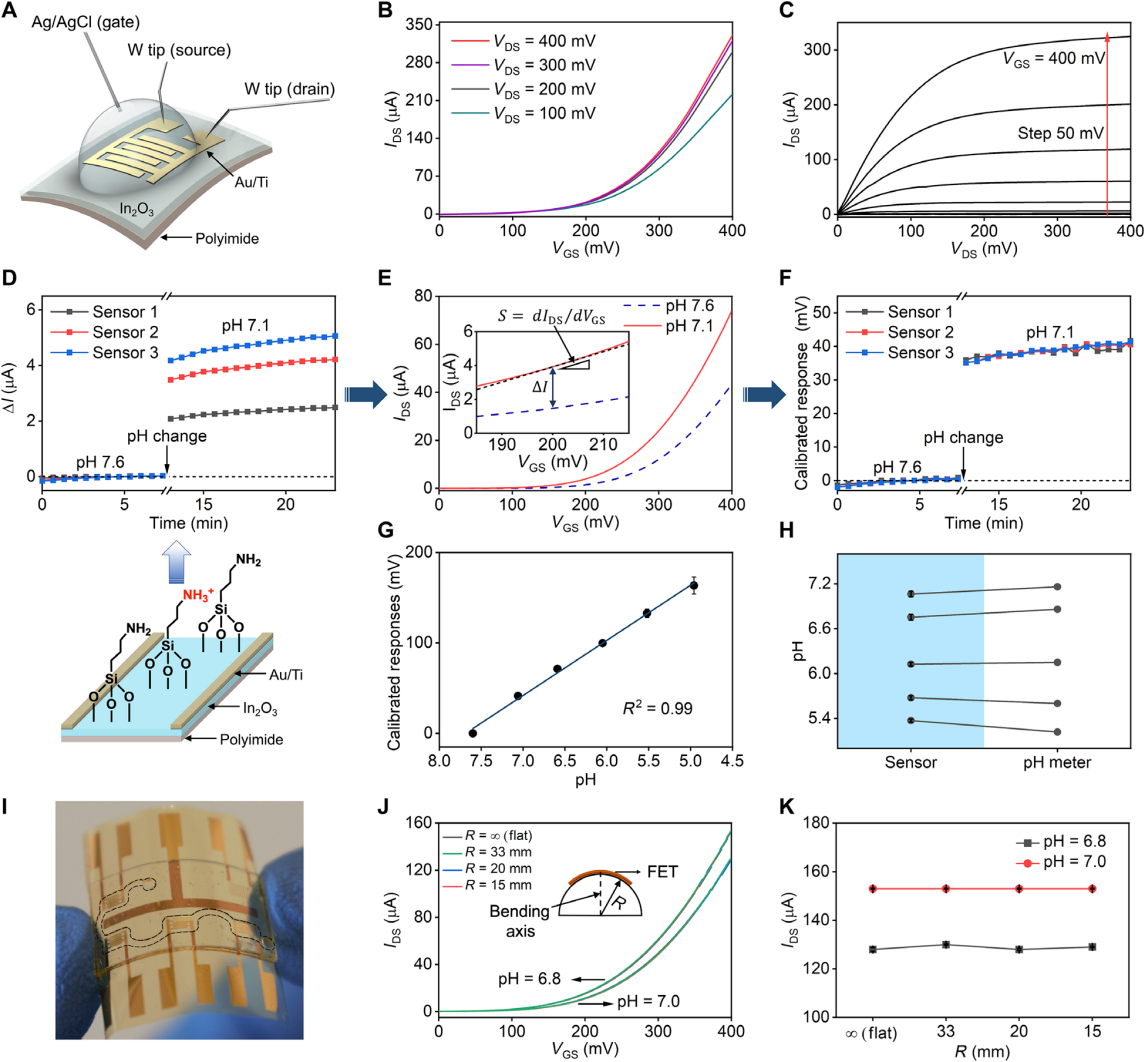
Flexible polyimide thin-film In_2_O_3_ FETs. (**A**) Schematic of the FET setup. A Ag/AgCl reference electrode was used as the solution gate. Current between the Au/Ti source and drain electrodes was recorded with tungsten (W) probes. (**B**) Transfer curves (*I*_DS_-*V*_GS_). The *V*_DS_ was varied from 100 to 400 mV in 100-mV increments; the *V*_GS_ was varied from 0 to 400 mV in 5-mV steps. (**C**) Transfer curves at different *V*_GS_ showing saturation behavior. The *V*_GS_ was varied from 0 to 400 mV with 50-mV steps. (**D**) Top: Real-time *I*_DS_ changes (Δ*I*) of FET-based pH sensors upon decreasing the solution pH from 7.6 to 7.1. Bottom: Channel surface charge perturbation mechanism. Primary amine groups of (3-aminopropyl)trimethoxysilane self-assemble on In_2_O_3_ and were protonated with decreasing pH (*V*_GS_ = 200 mV). (**E**) Calculation of FET calibrated responses with respect to individual FET transfer characteristics. Absolute sensor responses (Δ*I*) were divided by the slope (*S* = *dI*_DS_/*dV*_GS_, a gate-dependent component) to mitigate device-to-device variation. (**F**) Calibrated FET pH responses (corresponding to data in Fig. 2D; *V*_GS_ = 200 mV). (See also fig. S3). (**G**) Calibration curve for FET pH sensing (*N* = 3 FETs). (**H**) Unknown pH values determined by FET sensors versus a pH meter (*N* = 3; *V*_GS_ = 200 mV). (**I**) Photograph of a flexible FET array integrated with a tape-based microfluidic structure with the channel boundaries outlined (black dotted line). (**J**) Transfer curves from a representative FET sensor at pH 6.8 or 7.0 under different bending radii. The bending axis (*R*) is the shown in the inset. (**K**) The *I*_DS_ output of a FET sensor (*N* = 5 determinations for each pH condition and bending angle, *V*_GS_ = 400 mV). Error bars in (G), (H), and (K) are SEMs for each datum, which, in some cases, were too small to be displayed. *V*_DS_ = 400 mV for (D) to (K). Photo credit: Zhaoqing Wang, Yichao Zhao, UCLA.

Atomic force microscopy images indicated that thin (2 to 3 nm) In_2_O_3_ films were formed on polyimide with high uniformity over relatively large areas (e.g., wafer scale) (fig. S2). The roughness was minimal (root mean square roughness, 0.34 nm) and comparable to the roughness of In_2_O_3_ on Si (0.4 nm) (*36*). Polyimide films with FET arrays were delaminated from the underlying Si substrates for semiconductor analysis (Fig. 2A). Representative FET transfer and output characteristics are shown in Fig. 2 (B and C). Source-drain currents (*I*_DS_) were monitored over a range of drain voltages (*V*_DS_, 0 to 400 mV) and gate voltages (*V*_GS_, 0 to 400 mV) using a Ag/AgCl reference electrode for solution gate biasing.

We evaluated thin-film In_2_O_3_ FETs on flexible polyimide as pH sensors. The In_2_O_3_ was functionalized with (3-aminopropyl)triethoxysilane (APTES) diluted with trimethoxy(propyl)silane (PTMS) (1:9 v/v ratio) via self-assembly to form a pH-sensitive interface. Changes in hydrogen ion concentrations were detected via protonation/deprotonation of APTES amine tail groups (Fig. 2D), which alters surface charge to gate the underlying semiconductor. Since In_2_O_3_ is an n-type semiconductor, given the starting surface potential of our devices, increases in positive surface charge (i.e., increases in [H^+^], decreases in pH) increase *I*_DS_ (*39*, *40*).

Decreasing the pH of the solutions above FETs over a narrow physiological range from pH 7.6 to 7.1 produced measurable increases in *I*_DS_ (Fig. 2D). However, even considering differences in baseline currents at pH 7.6, pH-related changes in *I*_DS_ varied across three representative FETs. Device-to-device variation is a universal drawback for FET sensors that limits their accuracy. By implementing a previously reported self-referencing method (i.e., calibrated response) (*33*), we mitigated device-to-device variations (fig. S3).

We calibrated FET responses based on the *I*_DS_-*V*_GS_ transfer curves by normalizing absolute changes in *I*_DS_ to gate-voltage slopes at a given *V*_GS_ bias (200 mV) (Fig. 2E). Figure 2F demonstrates the use of this calibrated response method, where its application to absolute current measurements led to near identical FET-calibrated responses to pH change. As shown in fig. S3, pH-associated changes in calibrated responses calculated at different gate voltages produced similar results (*V*_GS_ = 150, 250, 300, or 350 mV) consistent with previous findings (*33*).

We next performed measurements over a broader pH range from 4.6 to 7.6. The FET calibrated responses were highly linear with respect to pH (*R*^2^ = 0.99) with negligible device-to-device variation (Fig. 2G). The practical utility of FET pH sensors was investigated by analyzing samples with unknown pH values and cross-correlating the results with measurements obtained using a laboratory pH meter. As shown in Fig. 2H, the FET pH values closely matched the pH meter values (*r* = 0.999, *P* < 0.001).

For wearable applications, we investigated the robustness of the underlying signal transduction mechanism of flexible FETs via pH sensing under mechanical deformation. Polyimide FETs were coupled to a tape-based thin-film microfluidic module (height, 170 μm; Fig. 2I) to introduce pH solutions when recording sensor responses under different bending radii. Responses to pH 6.8 or pH 7.0 solutions were determined under flat and bent conditions with different curvatures (*R* = 15, 20, or 33 mm). The FET transfer characteristics and current responses at both pH values were essentially identical regardless of the bending radii (Fig. 2, J and K, respectively). Furthermore, flexible In_2_O_3_ FETs showed consistent transfer characteristics even after 100 bending cycles (fig. S4) and have been previously reported to be stable after repetitive bending or crumpling with minimal mobility variations after 100 cycles (*30*).

### Development and validation of cortisol-aptamer-FET sensors

We identified a previously unknown DNA aptamer sequence (fig. S5A) that directly recognizes the human stress hormone cortisol using in vitro solution-phase systematic evolution of ligands by exponential enrichment (*41*, *42*). The solution dissociation constant (*K*_d_) of this cortisol aptamer was determined to be 500 nM via competitive fluorescence assays (fig. S5, B to E). We demonstrated the selectivity of the cortisol aptamer for the target (cortisol) versus chemically related biologically relevant nontargets (i.e., corticosterone, testosterone, and aldosterone; fig. S5, B and E). We investigated target-induced changes in aptamer secondary structural motifs using circular dichroism spectroscopy, as in our previous work (*27*, *28*, *43*). Upon target association, the cortisol aptamer showed a spectral shift and decrease in intensity in the major positive band (fig. S5F). These spectral changes suggest a partial disruption of a parallel G-quadruplex–like motif and a transition to a more extended single-stranded conformational state upon cortisol binding (*28*, *44*, *45*).

**Fig. 3.**
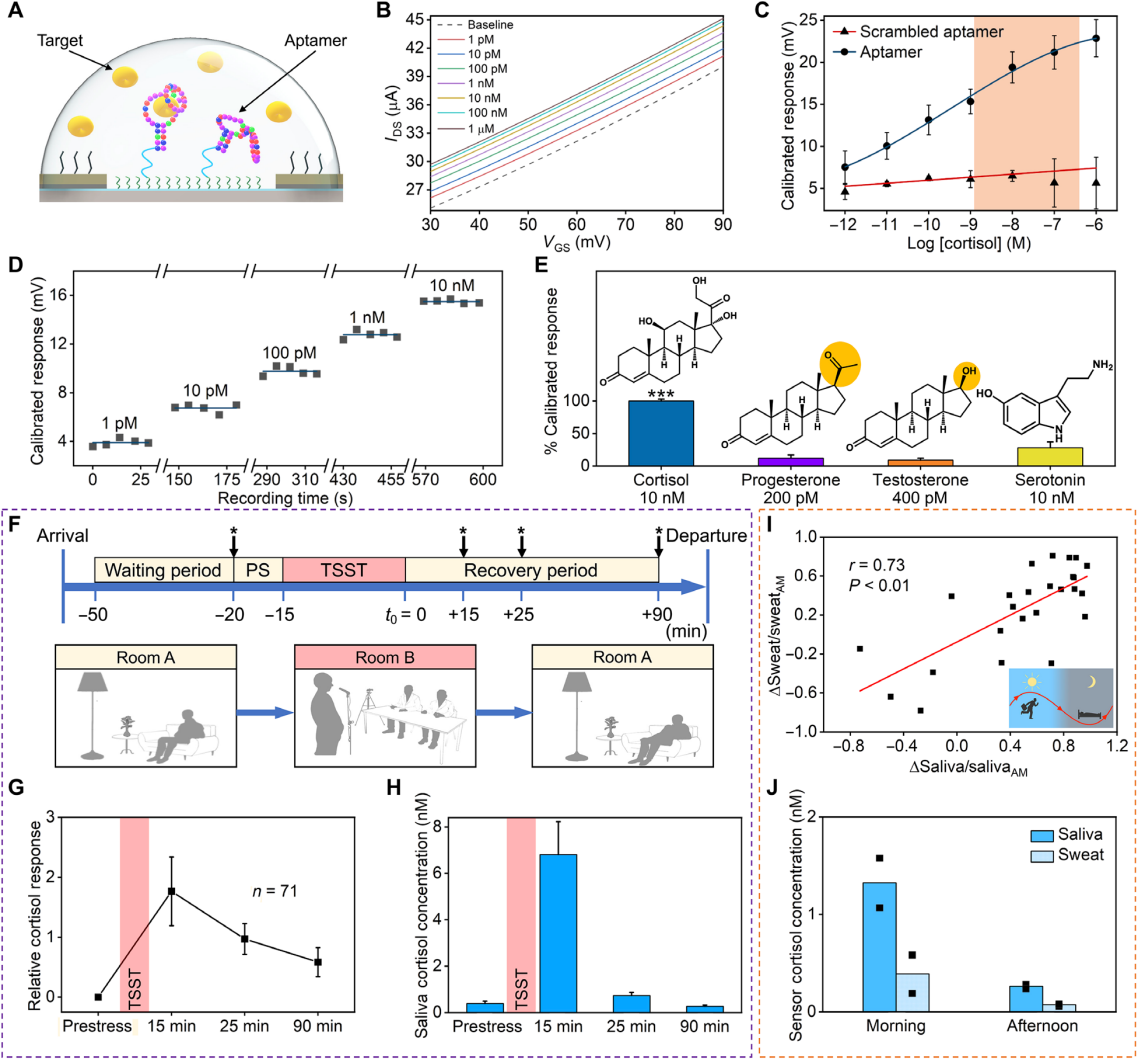
Biological applicability of aptamer-FET sensors. (**A**) Schematic of the aptamer-FET sensing mechanism. Cortisol-induced conformational changes occur in negatively charged aptamer phosphodiester backbones in conjunction with rearrangement of associated solution ions. (**B**) Aptamer-FET transfer curves in artificial sweat samples at varying cortisol concentrations. (**C**) Responses to cortisol for FETs functionalized with a cortisol aptamer (*N* = 3 FETs) or a scrambled sequence (*N* = 2 FETs) in artificial sweat. The physiologically relevant concentration range is highlighted. (**D**) Time-dependent cortisol-aptamer-FET responses to artificial sweat solutions with increasing cortisol concentrations. (**E**) Aptamer-FET responses to cortisol versus nontargets in artificial sweat illustrating negligible sensor responses to the latter. ***P < 0.001 versus nontargets (*N* = 3 FETs per target/nontarget). (**F**) The trier social stress test (TSST) protocol. The *t*_0_ is the reference time point corresponding to the stress period end. Starred arrows indicate saliva sampling times. PS, prestress. (**G**) Validation of the TSST protocol for eliciting cortisol responses. Cortisol was measured by standard laboratory assays. Four saliva samples were obtained at the time points indicated in (F) from 71 subjects. Relative cortisol responses are changes in cortisol with respect to individual prestress cortisol levels. (**H**) Cortisol response of a representative TSST participant measured by cortisol-aptamer-FET sensors (*N* = 3 replicates per time point; each measurement at a separate FET). (**I**) Morning (~9 a.m.) and afternoon (~5 p.m.) cortisol concentrations in sweat versus saliva samples from 17 healthy participants analyzed using an ELISA. The ΔSweat/Sweat_AM_ and ΔSaliva/Saliva_AM_ values were correlated and indicate decreases in cortisol levels in the afternoon with respect to the corresponding morning sample for each subject. (**J**) Morning and afternoon sweat/saliva cortisol levels from a representative subject measured using a cortisol-aptamer-FET. Dots represent measurements from the same sample on different devices. Error bars in (C), (E), (G), and (H) are SEMs for each datum.

To develop an aptamer-FET sensing interface, the cortisol aptamer with a thiol modification at the 5′ end was covalently immobilized on amino-silanized In_2_O_3_ FET channels using 3-maleimidobenzoic acid *N*-hydroxysuccinimide ester (MBS) as a cross-linker (fig. S6) (*28*). Aptamer-functionalized semiconductor channels translate target binding events into measurable surface charge perturbations originating from target-induced conformational changes in the negatively charged aptamer phosphodiester backbones in conjunction with rearrangement of associated solution ions (Fig. 3A). Changes in semiconductor surface charge manifest as changes in the effective *V*_GS_, and subsequently, *I*_DS_ and are quantified electronically in a label-free and reagentless manner.

Figure 3B illustrates transfer (*I*_DS_-*V*_GS_) curves from a representative cortisol-aptamer-FET sensor in response to different cortisol concentrations in artificial sweat. Cortisol-aptamer-FETs detected cortisol concentrations over six orders of magnitude (i.e., 1 pM to 1 μM; Fig. 3C). The on-FET *K*_d_ was determined to be ~30 pM. Similar sensing results were obtained in artificial saliva (fig. S7). Control experiments using FETs functionalized with a scrambled cortisol aptamer sequence composed of the same numbers of each nucleotide as the correct cortisol aptamer sequence but, with a different primary sequence and predicted secondary structure, produced negligible FET responses (Fig. 3C). Time-dependent cortisol-aptamer-FET responses to increasing concentrations of cortisol are shown in Fig. 3D. These data indicate that aptamer-FETs can be used to monitor dynamic changes in cortisol concentrations.

Aptamer-FET sensor responses are inherently nonlinear due to the properties of semiconductor gating. Therefore, we cannot describe sensor sensitivity and limits of detection as for conventional devices, such as electrochemical glucose sensors (*46*). Instead, we define the dynamic range (1 pM to 1 μM) as a critical parameter for cortisol aptamer FET biosensors, where 1 pM is the lowest practically detectable concentration. The lower detection limit of the cortisol dynamic range is similar to or lower than other reported cortisol sensing approaches (*19*, *22*, *35*). Our approach has the added benefits of being label-free and reagentless. The dynamic range covers the physiological range of cortisol in sweat and saliva (100 pM to 100 nM) (*47*–*49*).

We determined the selectivity of cortisol-aptamer-FETs by measuring responses to other closely structured steroid hormones (i.e., testosterone and progesterone) and the biogenic amine serotonin, all within their physiological concentration ranges in sweat and saliva (*50*–*52*). Cortisol-aptamer-FETs showed negligible responses to nontargets versus 10 nM cortisol, the estimated physiological concentration in sweat (Fig. 3E) (*19*). This aptamer-FET sensing approach can be applied, in principle, to other biomarkers in complex biological matrices by functionalizing individual FETs in arrays with different target-specific aptamers. To illustrate generalizability, we measured the target serotonin, which is also present in noninvasively retrievable biofluids such as sweat and saliva (fig. S8), using a previously isolated serotonin aptamer (*28*). Flexible polyimide serotonin-aptamer-FETs detected serotonin in artificial sweat over a large concentration range (10 fM to 100 μM; fig. S9), similar to the performance of serotonin-aptamer-FETs on Si or polyethylene terephthalate (PET) substrates (*28*–*30*).

We focused on cortisol detection, as many previous studies have demonstrated the clinical significance of cortisol in a variety of contexts (e.g., as informative of stress responses and circadian rhythm). Cortisol release is mediated by the hypothalamic-pituitary-adrenal axis, which has a central role in mobilizing the body to respond to physical and psychosocial stressors (*53*), as well as to disease and injury via inflammation (Fig. 1, A and B) (*54*). Normal cortisol levels follow a diurnal pattern where concentrations peak shortly after waking and then decline during the day (*55*).

Physiological and psychosocial stressors disturb circadian cortisol levels resulting in transient elevations (*55*, *56*). Cortisol levels vary greatly across people, and we anticipate that the ability to monitor individual cortisol levels will provide useful information for personalized medicine (*57*, *58*). Information on cortisol levels can be gleaned noninvasively on a person-by-person basis by making measurements in peripheral, easily accessible biofluids, such as saliva or sweat.

We utilized the TSST, a gold-standard laboratory procedure used to induce stress reliably in human participants (*56*) to establish stress-induced increases in salivary cortisol. The TSST consisted of (i) test environment acclimation, (ii) a prestress period when participants were informed about the upcoming task, (iii) a stress period where participants were asked to deliver a speech and then to respond verbally to a challenging arithmetic problem in the presence of two evaluators, and (iv) a recovery period (Fig. 3F). Saliva samples were collected from 71 healthy participants at four time points (i.e., prestress and 15, 25, and 90 min after stress). Salivary cortisol levels were quantified by a standard laboratory assay [i.e., liquid chromatography with tandem mass spectrometry (LC-MS/MS) or enzyme-linked immunosorbent assay (ELISA)].

Salivary cortisol concentrations peaked 15 min after the stress period and then declined over 75 min (Fig. 3G). We analyzed the saliva samples from a representative TSST participant using a cortisol-aptamer-FET device. The FET sensor measurements also revealed a cortisol peak 15 min after stress, followed by cortisol recovery to baseline 90 min after stress (Fig. 3H) in agreement with the aggregated trend demonstrated by the standard laboratory assays (fig. S10).

For wearable applications, establishing a saliva-sweat correlation is crucial as it enables leveraging existing knowledge of salivary biomarkers (*51*, *59*, *60*) as a foundation for future directions for sweat-based wearable applications. Hence, we performed a saliva-sweat correlation study. Saliva and sweat samples were collected from 17 healthy participants at two time points during the day (i.e., ~9 a.m. and ~5 p.m.). These times were selected as they are roughly the peak and nadir for diurnal variations in human cortisol levels. All samples were analyzed by ELISA. Most participants had higher saliva and sweat cortisol levels in the morning versus afternoon, in agreement with previous saliva cortisol studies (*57*, *58*). The correlation between salivary and sweat cortisol levels was 0.73 (Fig. 3I) supporting a correlation between salivary and sweat cortisol levels.

Cortisol-aptamer-FETs were used to determine diurnal variations in cortisol levels from saliva and sweat samples from a representative subject. The FET sensor responses showed elevated (morning) and decreased (afternoon) cortisol levels reflected in saliva and sweat samples (Fig. 3J), consistent with the observations made by analyzing the same samples by ELISA (fig. S10).

### Wireless aptamer-FET sensing system for wearable sweat analysis

**Fig. 4.**
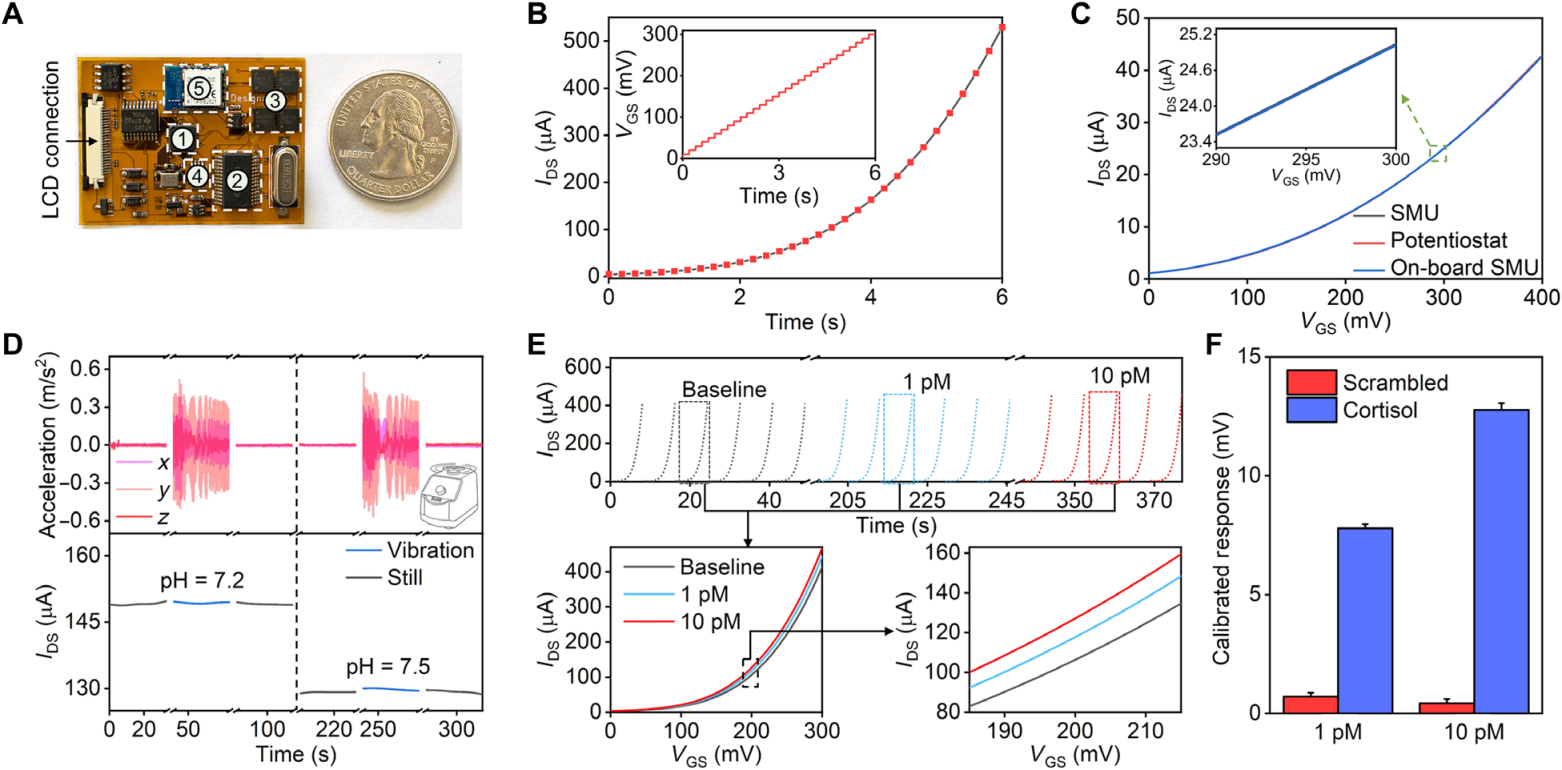
Integrated aptamer-FET sensing system with on-board SMU. (**A**) Photograph of the FPCB next to a U.S. quarter. The components are (1) MCU, (2) ADC, (3) potentiostat chip, (4) digital-to-analog converter, and (5) bluetooth. (**B**) Real-time sweep of *V*_GS_ and recording of *I*_DS_ to construct FET transfer curves measured by the SMU. (**C**) Comparison of FET transfer curves determined by a commercial SMU (Keithley 4200A-SCS, Tektronix, Beaverton, OR), a multichannel potentiostat (CHI1040C, CH Instrument, Austin, TX), and the on-board SMU. (**D**) Ex situ characterization of the FET sensing system with and without vortical vibration (microfluidic channel height: 170 μm). The recording was paused in between conditions to save sensor readouts and to distinguish scenarios. Vibrational acceleration profiles are presented on the top and sensor responses are displayed on the bottom when tested in pH 7.2 and pH 7.5 solutions. (**E**) A representative real-time recording of *I*_DS_ during *V*_GS_ sweeps (top) to track dynamic variations in FET transfer curves in response to blank (baseline), 1 pM, or 10 pM cortisol solutions in artificial sweat recorded by the on-board SMU. Bottom: Overlaid representative cortisol aptamer-FET transfer curves corresponding to the different solutions (higher-resolution plots on the bottom right illustrate that the transfer curves are distinguishable). (**F**) Comparison of cortisol aptamer-FET and scrambled oligonucleotide-FET (control) calibrated responses to 1 pM or 10 pM cortisol solutions in artificial sweat simultaneously recorded by the multichannel on-board SMU. Photo credit: Zhaoqing Wang, UCLA.

Detecting biologically relevant differences in cortisol in sweat using aptamer-FETs suggested utility for personal biomonitoring. These findings motivated the development of a wearable FET-array sensing system to track sweat cortisol and pH levels seamlessly. We included a FET functionalized with a scrambled cortisol aptamer sequence in the array to measure nonspecific responses. To illustrate versatility, we included a temperature sensor (fig. S11) (*4*, *30*). A representative multichannel flexible printed circuit board (FPCB) was designed to interface with the sensing array as illustrated in Fig. 4A.

The analog front-end was dedicated to FET sensor response acquisition and was implemented as a high-resolution SMU. Figure 4B illustrates a representative on-board SMU sweep of *V*_GS_ (with respect to a biased *V*_DS_) and recording of *I*_DS_ to acquire a FET transfer curve (6 s). We tested a commercial solid-state FET device (ADL110800) and compared the transfer curves obtained by our SMU with those captured by a commercial SMU (Keithley 4200A-SCS, Tektronix, Beaverton, OR) or a multichannel potentiostat (CHI1040C, CH Instruments, Austin, TX). The block diagrams of the standard laboratory instruments are shown in fig. S12. The transfer curves measured by all three instruments were closely matched (Fig. 4C), demonstrating the FET control/signal acquisition capability of our on-board SMU. We used an anisotropic conductive film (ACF) to establish electrical connection between the FPCB and the disposable sensing array forming a sensing system for reliable signal acquisition. For validation, we compared pH sensing using our FPCB/SMU to results obtained from a commercial multichannel potentiostat (fig. S13).

For on-wrist sweat applications involving arm movements, a tape-based thin-film microfluidic module was coupled to the FET sensing array. We evaluated the robustness of the signal acquisition by the integrated microfluidic sensing system in the presence of motion artifacts by wirelessly recording (via bluetooth) the real-time *I*_DS_ of a representative FET-based pH sensor under oscillatory motion (amplitude: ~3 m/s^2^ at 5 Hz, generated by a vortex mixer) (*6*, *61*). Characterization suggested a higher degree of signal robustness for a thinner microfluidic channel (fig. S14). Sensor responses exhibited negligible fluctuations (~1%) despite the motion (Fig. 4D), indicating that high-fidelity measurements were achieved by the complete system, in agreement with our recent studies (*6*).

We investigated simultaneous multichannel FET array response acquisition and the effectiveness of the calibrated response method to mitigate FET sensor variability using two FET-based pH sensor arrays each containing two FETs (fig. S15) Time-dependent *I*_DS_ was monitored at baseline (pH 7.4) and in response to pH decreases (pH 7.0 and 6.5) at FETs in each array. Baseline normalization resulted in a reduction in device-to-device variation from ~50 to ~30% (fig. S15, I and J). Using calibrated responses, variability across FETs was decreased to <10% (fig. S15, K and L) (*33*).

To test the capability of the sensor system to distinguish low levels of cortisol, we used a cortisol-aptamer-FET to track solution concentration changes. Real-time sweeps of *V*_GS_ and recordings of *I*_DS_ demonstrated that cortisol-aptamer-FETs detected cortisol as low as 1 pM (Fig. 4, E and F). As shown, the response time of the sensors is on the scale of seconds, while cortisol levels change in response to stress on the order of minutes to hours (Fig. 3, G and H). Leveraging the capability of the wearable system to measure from multiple aptamer-FETs simultaneously (i.e., from FETs functionalized with the correct cortisol aptamer or scrambled cortisol aptamer sequences that function as control sensors), we found that FETs functionalized with the scrambled oligonucleotide showed comparatively negligible responses (Fig. 4F).

**Fig. 5.**
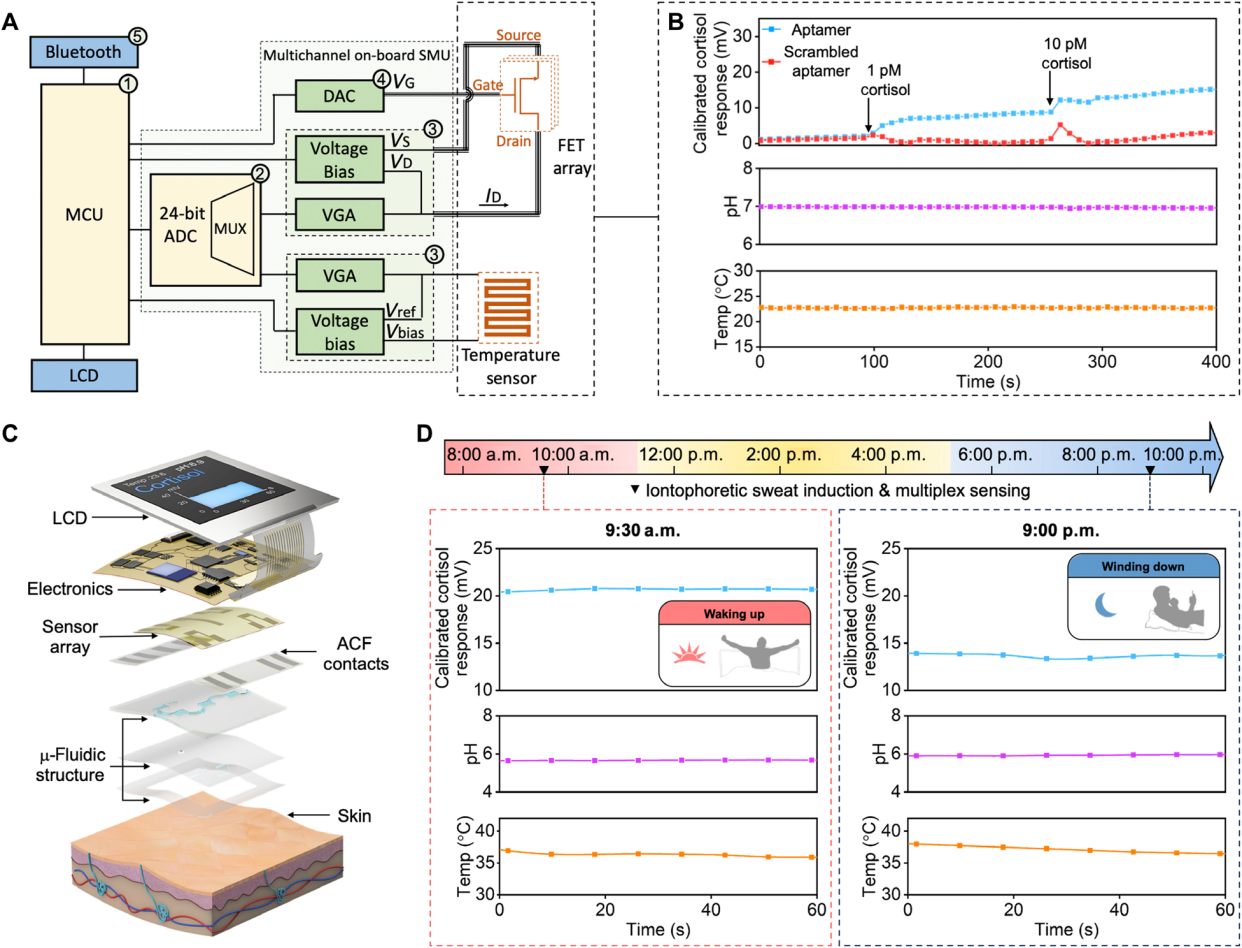
Wireless and wearable aptamer-FET sensing system for on-body sweat analysis. (**A**) Systems-level block diagram of the custom-developed wireless FPCB, equipped with an on-board SMU for programmable, multichannel, and high-resolution 24-bit analog-digital converter biosensing. Signals acquired and processed by the FPCB were displayed by a LCD and transmitted via bluetooth to a smartphone. (**B**) Representative real-time, multichannel ex situ measurements of cortisol solutions in artificial sweat, control, pH, and temperature captured by the on-board SMU. Responses at an active sensor functionalized with the correct cortisol aptamer are compared to responses at an inactive sensor functionalized with an incorrect (scrambled) sequence. (**C**) Expanded view of the wearable sensing system where the sensor array, microfluidic module, FPCB, and LCD components are integrated to form a multichannel biosensing smartwatch. (**D**) Real-time in situ monitoring of natural sweat cortisol, pH, and skin temperature from a healthy participant at two time points (9:30 a.m. and 9:00 p.m.) during routine daily activities with the multichannel biosensing smartwatch. Cortisol responses were obtained by subtracting the control channel reading (scrambled-oligonucleotide-FET) from the cortisol channel reading (cortisol-aptamer-FET).

Figure 5 (A and B) illustrates the integrated sensing capability for measuring cortisol (i.e., artificial sweat progressively spiked with 1 and 10 pM cortisol compared to a control sensor having a scrambled aptamer sequence that does not recognize cortisol) and simultaneous pH and temperature measurements. We incorporated a microfluidic module and a liquid crystal display (LCD) powered by a 110-mAh lithium polymer battery to produce a “smartwatch” (Fig. 5C). With a mobile phone application, the smartwatch acquired real-time measurements (i.e., cortisol, pH, and temperature) at set time intervals. We programmed the watch to take readings in the morning (9:30 a.m.) and evening (9:00 p.m.). To access sweat, iontophoretic stimulation was performed using a Macroduct Sweat Collection System (ELITechGroup Inc., Puteaux, France) on the volar surface of the forearm of the subject. The smartwatch was then placed on the stimulated area to collect, route, and analyze the secreted sweat. Figure 5D shows the real-time smartwatch recordings. The cortisol channel detected a decrease in the nighttime sweat cortisol level, in line with the typical circadian rhythm and observations from our ex situ correlation study (Fig. 3, I and J).

## DISCUSSION

We developed a fully integrated microfluidic sensing system capable of low concentration biomarker data acquisition that enabled the direct readout of a target biomarker (cortisol) concentration in a sample-to-answer manner (via dedicated electronics) suitable for wearable applications. Our approach simultaneously overcomes several important limitations associated with recently published sweat cortisol monitoring platforms (table S1) as it uses label-free detection, the sensing system is autonomous and wireless, the cortisol detection limits are ultralow (1 pM), and we validated sweat cortisol as a stress biomarker in a large clinical study. Readouts from standard methods versus aptamer-FET sensors revealed strong empirical correlations between cortisol levels in saliva and sweat samples in a pilot study. These results indicated the potential of sweat cortisol monitoring for translational applications, particularly considering an established body of knowledge related to salivary cortisol levels (*9*, *14*, *15*).

Aptamer-FETs are sensitive to environmental pH, because changes in local ion concentrations, including [H^+^], are detected by FETs (*30*). Thus, for translation, we developed an aptamer-FET array–based smartwatch equipped with high-resolution, multichannel biomarker data acquisition for the simultaneous, real-time, and seamless readout of cortisol levels, pH, and temperature. The generalizability of this FET sensing system enables adaptation to a wide range of target molecules using target-specific aptamers or other receptors (e.g., antibodies) (*62*, *63*) that facilitate measurable surface charge perturbations in response to target-receptor interactions. We are currently testing newly identified aptamers for additional stress biomarkers (e.g., epinephrine and norepinephrine). Once validated, these aptamers can be coupled with FETs in an array format to enable simultaneous quantification of multiple biomarkers to provide a more comprehensive view of the physiological status of users.

To enable translation of this technology into health and performance monitoring/optimization applications, dedicated and coordinated engineering and clinical efforts are required. To access target biomarker information on-demand in sedentary individuals, an iontophoresis interface will be needed to induce sweat secretion (*64*–*66*). For applications requiring continuous and prolonged biomarker sensing (e.g., athletic performance monitoring), sensor development efforts will need to focus on preserving sensor stability (e.g., antibiofouling strategies). In situ characterization of sweat secretion profiles (e.g., sweat rate, volume loss, etc.) will be helpful in normalizing readings for inter-/intra-individual physiological variations and gland activity variability.

Currently, our aptamer-FET biosensors are positioned for single-point measurements. However, aptamer-based biosensors have been successfully regenerated (*67*, *68*) and used for continuous analyte monitoring. We have shown here and in previous work (*28*, *30*) that gate voltage sweeps versus static gate-voltage bias produce different sensor behaviors. Although the sensing mechanism of aptamer-FETs relies on surface charge redistribution induced by target-induced changes in aptamer conformations, gate voltage also affects aptamer configurations. For example, gate voltage affects the local electronic environment of aptamers, and when changed (e.g., during sweeps), gate voltage can modulate aptamer conformations to low affinity states to release targets and, thus, to regenerate sensors. Further investigation of our sensing system will involve aptamer-FET measurements in larger numbers of clinical samples and continuous monitoring of cortisol fluctuations that involve decreases and increases. If large mechanical deformations of the sensing platform are anticipated, then further optimization will be needed to preserve the fidelity of data acquisition from both biosensor fabrication and system integration aspects.

From a clinical standpoint, given that sweat is a relatively underexplored biofluid, developing standard protocols will be advantageous (e.g., sweat-based TSST) to form the basis for large-scale, ambulatory, and longitudinal investigations centered on sweat-based biomarker studies. Accordingly, the advantages of our technology in terms of its ease of integration with wearable consumer electronics can be leveraged to facilitate these investigations (*69*). Large clinical datasets will enable physiological/psychobiological interpretations of sweat biomarker readings. These data can be contextualized to other user-specific static and dynamic information to render objective criteria for monitoring disease status (e.g., hormone imbalance disorders such as Cushing’s disease and Addison’s disease, assisting in the diagnosis of depressive disorders) and to provide personalized feedback to users to inform timely interventions (e.g., anxiety management via mindfulness or exercise) (*70*). For wearable applications, monitoring relative changes in biomarkers in an individual over time is more important for personalized feedback than absolute determinations. For example, one commercial wearable product, Oura ring (Oura Health, Oulu, Finland), monitors nightly average body temperature variations based on a baseline determined in each user, instead of absolute temperature values. Relative temperature monitoring based on modest individual fluctuations was found to be useful for menstrual cycle tracking (*71*). Through convergent efforts, noninvasive monitoring modalities will be established that can be leveraged to improve the productivity and health of individuals and society.

## METHODS

### Materials

All chemicals were purchased from Sigma-Aldrich Co. (St. Louis, MO) unless otherwise noted. Prime quality 4″ Si wafers (P/B, thickness 500 μm) were purchased from Silicon Valley Microelectronics Inc. (Santa Clara, CA). Oligonucleotides (table S2) were obtained from Integrated DNA Technologies (Coralville, IA). Indium(III) nitrate was purchased from Alfa Aesar (Thermo Fisher Scientific, Waltham, MA) and used as received. The SYLGARD 184 for producing polydimethylsiloxane (PDMS) wells was purchased from Dow Corning Corporation (Midland, MI). Water was deionized before use (18.2 megohm) via a Milli-Q system (Millipore, Billerica, MA). ACF (9703, Electrically Conductive Adhesive Transfer Tape; 50 μm) was purchased from 3M (Saint Paul, MN).

### Aptamer selection and characterization

The cortisol aptamer selection was carried out as per previously published methods (*28*, *41*, *42*) with modifications to the target concentration and choice of nontargets (table S3 and fig. S18). The method was based on selection of oligonucleotide sequences that favor solution target association (elution) versus capture strand binding (retention). Oligonucleotides used in the selection process were (i) an N36 random library: 5′-GGA GGC TCT CGG GAC GAC- (N_36_)-GTC GTC CCG CCT TTA GGA TTT ACA G-3′, (ii) a biotinylated column immobilizing capture strand: 5′-GTC GTC CCG AGA GCC ATA/3BioTEG/, (iii) a forward PCR primer: 5′-GGA GGC TCT CGG GAC GAC-3′, (iv) a reverse primer: 5′-CTG TAA ATC CTA AAG GCG GGA CGA C-3′, and (v) a biotinylated reverse-primer: /5Biosg/ CTG TAA ATC CTA AAG GCG GGA CGA C. See also table S2.

Standard desalted oligonucleotides were used for the library and primers. Modified oligonucleotides (e.g., biotinylation, fluorophore conjugates) were purified by the manufacturer. All oligonucleotides were dissolved in nuclease-free water and stored −20°C. Polymerase chain reaction (PCR) amplifications were run with 1 cycle at 95°C for 2 min, *N* cycles at 95°C for 15 s, 60°C for 30 s, and 72°C for 45 s, and 1 cycle at 72°C for 2 min. In most cases, PCR was carried out over 11 ± 1 cycles. We used commercially available phosphate-buffered saline (PBS) [Corning catalog no. 21-040-CV; 154 mM NaCl, 5.6 mM Na_2_PO_4_, and 1.058 mM KH_2_PO_4_ (pH 7.3 to 7.5)] with additional 2 mM MgCl_2_ for most selection rounds. Four rounds were carried out with an NxStage pureflow solution (RFP402, NxStage Medical, Lawrence, MA) in place of PBS buffer (table S3). Candidate aptamer sequences identified by selections are shown in table S4.

The cortisol aptamer sequence (table S2) was modified with fluorescein at the 5′-end (5′/56-FAM/CTC TCG GGA CGA CCG GTC TGG GGA CCC TGT CTG GGT GTG TGG GTA GTA GGT CGT CCC-3′). The quencher strand was labeled with dabcyl at the 3′-end (5′- GGT CGT CCC GAG AG/3Dab/-3′). The aptamer-to-quencher ratio (1:5) and assay conditions were as previously described (*42*). The cortisol aptamer *K*_d_ was determined as described by Hu *et al.* (*72*) in PBS with 2 mM MgCl_2_ (fig. S5B).

We used a thioflavin T (ThT) assay to investigate aptamer specificity (*73*). Final concentrations in the incubation solutions were aptamer (400 nM), ThT (4 μM), and target or nontargets (0 to 10 μM) (fig. S5C). The aptamer was incubated in 95°C PBS for 5 min (1.6 μM) and cooled to room temperature over 30 min. Aptamer and ThT (16 μM in PBS) were mixed (1:1 ratio) and incubated for 40 min. Targets or nontargets (2× final concentrations in PBS) were added to each oligonucleotide/ThT sample solution. Target/nontarget concentrations were tested in triplicate in a final volume of 135 μl. Fluorescence measurements were performed using a Molecular Devices Flexstation II plate reader (Molecular Devices, San Jose, CA) with 425-nm light for excitation and recording emission at 495 nm.

For circular dichroism determination of aptamer secondary structure, aptamer and target concentrations were 1 μM in artificial sweat. Aptamers were thermally treated as described below. Spectra were collected on a JASCO J-715 circular dichroism spectrophotometer (Jasco Products Company, Oklahoma City, OK) at room temperature. Four scans were acquired per sample with 0.5-nm resolution, 1.0-nm bandwidth, a 4-s response time, and a scan rate of 20 nm/min. Scans are averages of four instrumental scans and representative of three replicates per condition. Scans in artificial sweat without targets were subtracted as background.

### FET fabrication and functionalization

Polyimide films were fabricated using a PI-2611 solution (HD MicroSytems, Parlin, NJ). The PI-2611 solution was used as received and was spin-coated onto Si wafers directly at 3000 rpm for 30 s. The film was baked at 150°C for 90 s, followed by thermal annealing at 350°C for 30 min in an oven. The polyimide film thickness was ~7 μm as per the technical information provided by HD MicroSystems for PI-2611 and was confirmed using a profilometer (Dektak 6M profilometer, Bruker, Billerica, MA).

Aqueous solutions (0.1 M) of indium(III) nitrate hydrate [In(NO_3_)_3_•xH_2_O, 99.999%] were then spin-coated (3000 rpm) for 30 s on flexible polyimide substrates or heavily doped silicon wafers (Silicon Valley Microelectronics, Santa Clara, CA) with 100-nm-thick thermally grown SiO_2_ layers (*32*, *36*). After coating, substrates were prebaked at 150°C for 10 min followed by thermal annealing at 350°C for 4 hours (*37*, *38*). Surface roughness of In_2_O_3_ was measured using an atomic force microscope (Bruker Dimension FastScan, Billerica, MA) and calculated as the root mean square of peaks and valleys in each measured topographic image (fig. S2B) using Nanoscope Analysis (Bruker, Billerica, MA). Patterning of In_2_O_3_ was by photolithography followed by dry etching using a STS advanced oxide etcher (Surface Technology Systems plc, Newport, United Kingdom). Interdigitated source and drain electrodes (1500-μm length, 80-μm width, 10-nm Ti, and 30-nm Au) were patterned by photolithography with metal deposition by electron beam evaporation (*28*). After fabrication, the polyimide was delaminated and cut using a razor blade.

FETs were functionalized using previously reported protocols (*27*, *28*). Specifically, APTES and PTMS (1:9 v/v ratio) were self-assembled on In_2_O_3_ using vapor-phase deposition. Solutions and devices were placed in a water bath at 40°C for 1 hour followed by baking on a hot plate at 80°C for 10 min. Devices were then incubated with 1 mM 1-dodecanethiol in ethanol for 1 hour to passivate the Au source and drain electrodes. The FETs for pH sensing were used without further modification.

To fabricate aptamer-functionalized FETs, silanized FETs were rinsed with ethanol and immersed in 1 mM MBS, which was dissolved in a 1:9 (v/v) mixture of dimethyl sulfoxide and PBS [(pH 7.4) Gibco, Thermo Fisher Scientific Inc., Waltham, MA] for 30 min. In parallel, thiolated DNA aptamers were prepared by heating at 95°C for 5 min in nuclease-free water followed by rapid cooling in an ice bath and a return to room temperature. The MBS-modified In_2_O_3_ surfaces were rinsed with deionized water and immersed in 1 μM thiolated DNA aptamer solutions overnight (>12 hours) for aptamer immobilization. The MBS cross-linked amine-terminated silanes with thiolated DNA aptamers. Before measurements, aptamer-FET sensors were rinsed with deionized water and blown dry with N_2_ gas.

A scrambled sequence with the same numbers and types of nucleotides as the correct aptamer sequence but with a pseudo-random order was designed to investigate nonspecific aptamer-target recognition on FETs (table S2). The scrambled sequence was selected on the basis of modeling (mfold: http://unafold.rna.albany.edu/?q=mfold) to adopt a different secondary structure compared to the correct sequence.

### FET biosensing

For pH sensing, each amine-functionalized FET was immersed in PBS with a Ag/AgCl reference electrode (SDR2; 2-mm diameter; World Precision Instruments Inc., Sarasota, FL), which acted as the gate electrode (liquid gate). Sensor measurements were performed using a multichannel electrochemical workstation (CHI1040C, CH Instrument, Austin, TX). Multichannel input was used to obtain transfer curves. To achieve gate-source sweep voltage biasing (*V*_GS_), the Ag/AgCl electrode (channel 1) had an applied linear sweep from 0 to +0.4 V at 10 mV/s. The counter and reference electrode connectors of channel 1 were connected to the source electrode of each FET. To achieve a constant drain-source bias voltage (*V*_DS_), the drain electrode was connected to the working electrode connector of channel 2 and a constant potential was applied (0.4 V).

Five overlapping transfer curves at each pH were averaged. Calibrated responses (*V*_GS_ = 200 mV) were calculated to minimize device-to-device variations as described in Results and the Supplementary Materials (*33*). The accuracy of the FET pH sensors was validated by comparing the measured results with corresponding measurements obtained using a standard pH meter (Themo Fisher Scientific AE150).

For aptamer-FET measurements, PDMS wells were placed over individual FETs to contain sensing solutions. Artificial saliva (1700-0303, Pickering Laboratories Inc., Mountain View, CA) or artificial sweat (I2BL-0011, Pickering Laboratories) were used as electrolyte solutions (table S5). The Ag/AgCl reference electrodes were placed in the sensing solutions above FETs. Sensor measurements were performed using a manual analytical probe station (Signatone, Gilroy, CA) equipped with a Keithley 4200A-SCS semiconductor parameter analyzer (Tektronix, Beaverton, OR). Transfer curves were obtained by sweeping *V*_GS_ (0 to 400 mV at 5-mV steps, *V*_DS_ 10 mV). Five overlapping transfer curves were averaged for each target or nontarget concentration. Calibrated responses to minimize device-to-device variations were calculated at *V*_GS_ = 100 mV. Signals acquired by aptamer-FETs (i.e., receptor target binding) are nonlinear by nature (i.e., described by a Langmuir binding isotherm) and are conventionally represented on a logarithmic scale (*27*, *28*, *30*, *36*, *74*, *75*). Minimal leakage current from the reference electrode was verified (fig. S16). Any FETs that did not stabilize or showed poor transfer curve characteristics were not used.

### FET bending

A polyimide-FET pH sensor was interfaced with a tape-based thin-film microfluidic structure and connected to a potentiostat with the aid of ACF. The microfluidic structure was first fixed on a flat surface and injected with PBS (pH 7.0 and pH 6.8 for two sets of tests) through the inlet of the microfluidic structure. Transfer curves during flat conditions were recorded. Next, sensors were conformally attached to the surfaces of cylinders with radii of 33, 20, or 15 mm. Transfer curves for each bending condition were determined. The FET sensor gate was driven through an on-chip Ag/AgCl reference electrode, which was fabricated by depositing Ag/AgCl ink (Ercon, Wareham, MA) on the electrodes and heating the modified electrodes at 80°C for 10 min.

### Trier Social Stress Test

Psychological stress was produced by the TSST to induce changes in cortisol levels (*56*). Saliva samples for this study were provided from a parent study (*N* = 71) conducted in the Department of Psychology at the University of California, Los Angeles [UCLA; Institutional Review Board (IRB) no. 14-001311]. Participants were at least 18 years old, identified as Black/African American or Hispanic/Latino(a), reported a household income less than or equal to 200% of the federal poverty line, and were fluent in English (for the purposes of delivering the speech task during the laboratory visit). Exclusion criteria (due to incompatibility with study methods or eating outcomes) included history of an eating disorder, currently adhering to a strict diet, nut or food allergies, current major illness, injury, or mental health diagnosis. Additional exclusion criteria related to incompatibility with salivary cortisol sampling included metabolic or endocrine disease (*76*), chronic asthma (*77*), history of substance abuse (*78*), current use of opiates, steroids (other than inhaled steroids) or antipsychotic medications (*78*), or postmenopausal status (*78*).

Participants were scheduled for a laboratory session between the hours of 2:00 p.m. and 5:00 p.m. to control for the diurnal pattern of cortisol (*56*–*58*). The TSST involved two main tasks performed in front of an evaluative audience: (i) public speaking and (ii) mental arithmetic. To summarize the protocol briefly, participants were informed about the upcoming tasks and were given 3 min to prepare. They then performed a 5-min speech where the goal was to convince a panel of two evaluators, clad in laboratory coats, that they were the best candidate for a hypothetical job opening. Each speech was videotaped; participants were told their performances would be behaviorally evaluated. Throughout the speech, the evaluators were trained to gaze at participants with neutral faces and at regular intervals, interrupt with sentences such as, “What are your major shortcomings or weaknesses?”

The 5-min mental arithmetic portion required participants to start with the number 2935 and serially subtract by 7 and then, after 1 min, by 13. Each time a participant made an error, they were instructed to start over at 2935, and the evaluators were trained to deliver lines such as, “This is just subtraction, try to focus,” throughout the task. The TSST was followed by a 90-min recovery period where the participants watched a neutral documentary.

Saliva (passive drool) was collected at baseline (prestress) and 15, 25, and 90 min after stress. Participants were asked to rinse their mouth with water before saliva collection. At the end of the session, all participants were debriefed and compensated with either course credit or $50. Saliva samples (2 ml) were stored at −20°C before analysis. Saliva samples were centrifuged at 10,000 rpm for 20 min before cortisol measurements. The samples were analyzed by aptamer-FETs or standard methods (ELISA or LC-MS/MS).

### Diurnal saliva/sweat sample collection

Human subject experiments were conducted in compliance with protocols approved by the IRB at UCLA (IRB no. 17-000170). All participants gave written informed consent before participation in the study. A pilot study (*N* = 17) was conducted for investigation of cortisol saliva-sweat correlation and validation of cortisol aptamer-FET sensors. Healthy participants were recruited for saliva and sweat collection. Cortisol production undergoes diurnal variation with the highest levels present after waking and the lowest levels present around midnight (*57*, *58*). Saliva and sweat sample pairs were collected in the morning (~9:00 a.m.) and afternoon (~5:00 p.m.).

On the day of sample collection, participants were told to report to the laboratory within 1 hour of waking and to refrain from food intake at least 1 hour before sample collection. To collect sweat following a standard protocol, the volar surface of each participant’s forearm was cleaned with deionized water and ethanol, followed by sweat gland stimulation using iontophoresis for 5 min. Participants were asked to rinse their mouths with water before saliva collection. Saliva was collected via passive drool after sweat stimulation. Samples were stored at −20°C until analysis.

### Saliva and sweat sample laboratory analyses

Salivary Cortisol ELISA RUO (research use only, SLV2930R, DRG Inc., Springfield, NJ) or LC-MS/MS was used for the quantitative determination of cortisol in human saliva or sweat. Samples were diluted 1- to 10-fold in sample buffer before analysis. For ELISA, the assay for cortisol was performed using the manufacturer’s protocol. Sensors were tested in artificial saliva (fig. S7 and table S5), which does not contain all species in authentic saliva (e.g., urea). Sensors were also tested in real saliva samples (Fig. 3, H and J), which contains urea. Artificial saliva was acquired from Pickering Laboratories Inc. (Mountain View, CA) and was formulated according to standard methods (Institut für Normung 53160).

For LC-MS/MS with multiple reaction monitoring (MRM) analyses, protocols for each biomarker were developed similar to previous work (*49*, *79*, *80*). Human saliva or sweat samples were centrifuged at 14,000 rpm for 10 min, and the supernatants were used for analysis. A solid-phase extraction (SPE) technique was used to extract cortisol or serotonin from standard solutions and human saliva or sweat samples (SPE cartridges: Oasis HLB, Waters Corporation, MA). Deuterated cortisol (cortisol-*d*4 (9, 11, 12, 12-*d*4) or serotonin (serotonin-*d*4 hydrochloride) was used as the internal standards for quantification of cortisol and serotonin, respectively.

An Agilent 1200 series high performance liquid chromatograph (Agilent Technologies, Palo Alto, CA) equipped with an HTS PAL autosampler (CTC Analytics, MN) was coupled to an API 4000 triple quadrupole MS (Sciex, ON, Canada) for MRM experiments. A Zorbax 300 SB-C18 column (0.5 internal diameter × 150-mm length, 5-μm particle size; Agilent Technologies) was used for separation. Solvent A was water with 0.1% formic acid; solvent B was acetonitrile with 0.1% formic acid. For cortisol analysis, the flow rate was 400 μl/min with the following gradient: 10% B (0.0 to 0.5 min), 10 to 90% B (0.5 to 5.5 min), 90% B (5.5 to 8.5 min), 90 to 10% B (8.5 to 9.0 min), and 10% B (9.0 to 11.0 min). For serotonin analysis, the flow rate was 400 μl/min with the following gradient: from 5 to 20% B (0.0 to 3.0 min), 20 to 90% B (3.0 to 5.5 min), 90% B (5.5 to 8.5 min), 90 to 5% B (8.5 to 9.0 min), and 5% B (9.0 to 11.0 min). Sample vials were maintained at 4°C in the autosampler tray. A 20-μl aliquot of each sample was injected onto the column.

The instrument was operated in the MRM mode with the following mass/charge ratio transitions: 363.3 → 121.1 for cortisol (fig. S17), 367.3 → 121.1 for cortisol-*d*4, 177.2 → 160.0 for serotonin (fig. S8), and 181.2 → 164.2 for serotonin-*d*4. Peak area ratios of the analytes (cortisol or serotonin) to their respective internal standards were plotted as a function of analyte concentration to construct calibration curves. Analyte concentrations in human saliva or sweat samples were determined on the basis of peak area ratios relative to internal standards and calibration curves. For measurements with each aptamer-FET, the baseline current (artificial saliva or sweat) was collected, and then a sample of diluted human sweat or saliva was added so that the final cortisol concentration in the PDMS well was theoretically ~10 pM (assuming ~10 nM cortisol in each sample) and sensor responses were collected.

### Wireless wearable system design

A dedicated analog, front-end unit was designed and incorporated onto the FPCB to acquire FET transfer curves. Briefly, programmed by the microcontroller unit (MCU) and with the aid of a digital-to-analog converter (DAC), the gate voltage (*V*_G_) was periodically swept over the desired range with optionally adjustable biased source and drain voltage levels (*V*_S_ and *V*_D_). The resulting FET *I*_DS_ was converted to voltage using a transimpedance amplifier with a programmable feedback resistance, effectively implementing a variable gain amplifier (VGA). Similar VGA and voltage biasing configurations were adopted to acquire temperature sensor responses manifested as changes to measured resistance. The output for each of the sensing channels was converted to the digital domain and relayed to the MCU using a high-resolution analog-to-digital converter (ADC) with a multiplexer front.

In our design, the DAC (DAC8552, Texas Instruments) was connected to the gate of each FET sensor to perform *V*_GS_ sweeps (0 to 400 mV, 10 mV steps at 200-ms intervals). The source and drain electrodes of each FET were biased (400 mV) with a potentiostat chip (LMP91000, Texas Instruments, Dallas, TX). The current response (*I*_DS_) between the working electrode pin of the potentiostat chip was amplified and converted to voltage by the built-in transimpedance amplifier (programmable TIA, gain: 2.75 kilohm). The analog voltage signal output was converted to the digital domain by a multichannel 24-bit ADC (ADS1256, Texas Instruments) chip at a sampling rate of 200 Hz. A microcontroller chip (Atmega328, Microchip Technology, Chandler, AZ) was used to control the output voltage of the DAC and to collect the readout signal from the ADC by serial peripheral interface communication, where each data point was averaged over 10 readings.

This circuit board communicated wirelessly and bilaterally with a mobile application user interface on a cell phone via an on-board bluetooth module (AMB2621, Wurth Elektronik, KG, Germany). The acquired and processed sensor outputs were displayed and plotted on a 1.44″ color LCD display (SF-TS144C-9082A-N, Shenzhen SAEF Technology, Shenzhen, China). The entire system was powered by a 110-mAh Li-ion battery (PRT-13853, SparkFun Electronics, Boulder, CO). A smartwatch case was used to hold the sensor array, microfluidic structure, and electronic modules, as well as the battery. The integrated smartwatch was adhered to the wrist with double-sided tape.

### FPCB validation

A cortisol aptamer-FET sensor was immersed in a PBS solution and connected to the FPCB. The FET source and drain electrodes were biased at 400 mV. The gate voltage was swept following a staircase waveform from 0 to 400 mV (10-mV step increments at 200 ms). For each step, 10 readings were sampled and averaged to obtain the *I*_DS_ corresponding to each applied *V*_GS_. The *I*_DS_ values were used to construct the transfer curves pertaining to each *V*_GS_ sweep. A solid-state FET (ALD110800, Advanced Linear Devices Inc., Sunnyvale, CA) was characterized by the FPCB module, potentiostat, and SMU sequentially to verify the FPCB signal acquisition functionality.

### Multiplexed measurements with a custom-developed circuit board

For multiplexed pH measurements, two devices (each containing two FET pH sensors) were used. Commercial Ag/AgCl reference electrodes were used to drive the gates. Each device was immersed in its own beaker with a PBS solution. The four pH sensors were connected to the multichannel on-board SMU for biasing and data recording. Hydrochloric acid was spiked twice in both beakers. Transfer curves for all sensors under different pH conditions were recorded in real-time. The pH values in both beakers were also recorded by a standard pH meter simultaneously. For ex situ multiplexed measurements with the board, a PDMS well was placed on a polyimide-based FET sensor array, which contained one cortisol sensor, one control sensor (with the scrambled cortisol aptamer), one FET pH sensor, and a temperature sensor. An on-chip Ag/AgCl electrode was used to drive the gate and fabricated as described above. The custom FPCB was connected to the sensor array to provide biasing. Cortisol solutions were spiked into the PDMS well to change the cortisol concentration to 1 and 10 pM sequentially.

### Characterization of the wireless FPCB module

A polyimide-FET pH sensor was interfaced with a tape-based thin-film microfluidic device (~170 μm for each layer) and connected to a custom-developed FPCB with the aid of ACF. The FPCB-connected sensor was then fixed onto a vortex mixer (Thermo Fisher Scientific, Waltham, MA) together with an accelerometer (on a smartphone). Artificial sweat (pH 7.2) was injected through the inlet of the microfluidic device to fill the entire structure. Vortical vibrations were introduced by the mixer (5 Hz). Sensor signals were acquired and transmitted wirelessly (via bluetooth) and recorded on a cellphone. Next, artificial sweat pH 7.5 was injected into the microfluidic device to replace the previous solution. The same characterization process was then conducted.

### Wearable FET sensing system fabrication

Each FET sensor array was adhered onto the electrical contacts located on the back of the smartwatch using ACF. The FET sensor array was embedded within a tape-based thin-film microfluidic device. Microfluidic channels were created by laser cutting 2D patterns on double-sided tape (~170 μm, 3M Science, MN; VLS2.30; Universal Laser System, AZ). Outlet features were created by laser patterning holes on PET (~100 μm; MG Chemicals, Surrey, BC, Canada) to facilitate an ejection path for sampled biofluids. The channel width was 200 μm, and the sensing chamber dimension was 3 mm by 1.5 mm. The microfluidic device/module was then aligned and assembled by attaching the patterned PET layer to the patterned double-sided tape. It typically took 5 to 15 min for sweat to fill the microfluidic channels after sweat gland iontophoretic simulation on a 1.2-cm^2^ area of skin. We have used similar sweat harvesting strategies for biofluid management and biomarker analysis (e.g., pharmaceuticals and metabolites) (*65*, *66*, *69*).

The power consumption of the smartwatch was strongly dominated by the LCD, which had a power dissipation of 0.288 W. The LCD as a heat source was isolated from the sensor by the electronic device and the flexible PCB board. The gap between the LCD and sensor was 3.3 mm. This gap protected the sensor from temperature disturbances. The temperature change on the sensor surface after 10 min of continuous smartwatch operation increased 0.9°C (from 23.4° to 24.3°C), which should not affect aptamer-FET sensing. We integrated a temperature sensor next to the aptamer-FET array. In future studies, we can investigate the effect of small temperature changes on aptamer-FET responses, and the integrated temperature sensor can be used for correction if there is any response of aptamer-FETs to temperature variation.

Before on-body sweat multiplexed measurements, the assembled device was attached to the wrist skin of a healthy participant via double-sided tape, and FET sensor baselines were recorded in artificial sweat for self-calibration. To induce sweat iontophoretically, the target stimulation area of the skin was first cleaned with deionized water and ethanol, followed by 5 min of iontophoretic sweat gland stimulation (with pilocarpine-loaded hydrogels, Pilogel) using a Macroduct Sweat Collection System (ELITech Group, Puteaux, France). Measurements were conducted at 9:00 a.m. (1 hour after awakening) and 9:30 p.m. to capture peak and nadir cortisol levels, respectively. The subject refrained from food intake for at least 1 hour before each test to avoid confounding effects on body cortisol production. The responses from control sites were subtracted from responses at cortisol sensing sites.

To communicate wirelessly with the FPCB module, an illustrative Android smartphone application was developed (fig. S19). The application provided a graphical user interface to execute a range of functionalities, including setting the desired operational modes and data display and storage. The Android application was designed to establish communication with the wearable module upon startup. In our implementation, the user input was read with the aid of touchscreen-activated buttons and relayed to the FPCB through the communication of predefined integer values (each value mapped to the desired operation) using Bluetooth. The corresponding commands were received and executed at the microcontroller level. Once communication was established, the user could observe the real-time status of the cortisol, temperature, and pH responses. The real-time and filtered sensing results were then recorded and timestamped in a separate text file on the phone. After the sensing period, the data were uploaded and stored automatically in a Google Cloud Storage bucket.

### Statistics

Statistical analyses were carried out in OriginPro (2021, Northampton, MA). Correlations for FET pH sensing versus pH meter determinations in Fig. 2H, saliva versus sweat cortisol level correlation in Fig. 3I, and correlations of cortisol levels by aptamer-FETs versus standard laboratory assays (fig. S10) were analyzed by Pearson correlations. Data for Fig. 3E were analyzed by one-way analysis of variance (ANOVA) followed by post hoc Dunnett’s multiple comparisons.

## Supplementary Materials

### This PDF file includes:

Figs. S1 to S19 Tables S1 to S5 References
