# Supplementary material for: Wearable aptamer-field-effect transistor sensing system for noninvasive cortisol monitoring
Source: Sci Adv. 2022 Jan 5;8(1):eabk0967. doi: 10.1126/sciadv.abk0967 (PMC8730602; doi:10.1126/sciadv.abk0967)
Supplement: Supplementary file 1 — Figs. S1 to S19 Tables S1 to S5 References [file sciadv.abk0967_sm.pdf]

Science Advances
AAAS

Supplementary Materials for

Wearable aptamer-field-effect transistor sensing system for noninvasive cortisol monitoring

Bo Wang, Chuanzhen Zhao, Zhaoqing Wang, Kyung-Ae Yang, Xuanbing Cheng, Wenfei Liu, Wenzhuo Yu, Shuyu Lin, Yichao Zhao, Kevin M. Cheung, Haisong Lin, Hannaneh Hojaiji, Paul S. Weiss, Milan N. Stojanović, A. Janet Tomiyama, Anne M. Andrews*, Sam Emaminejad*

*Corresponding author. Email: aandrews@mednet.ucla.edu (A.M.A.); emaminejad@ucla.edu (S.E.)

Published 5 January 2022, *Sci. Adv.* **8**, eabk0967 (2022)
DOI: 10.1126/sciadv.abk0967

This PDF file includes:

Figs. S1 to S19
Tables S1 to S5
References

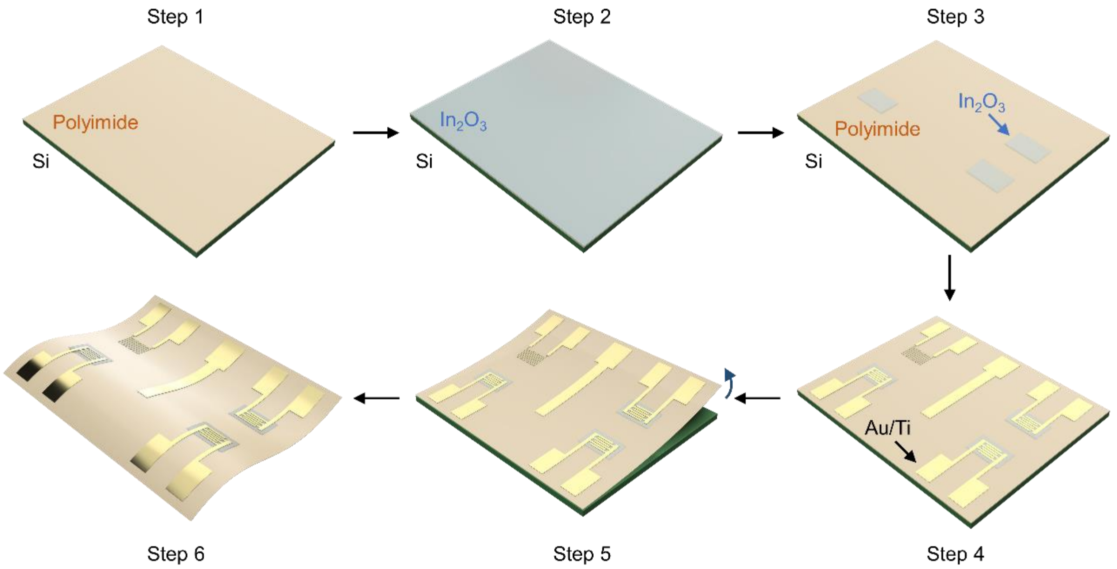

figure S1. Schematic illustration of flexible sensor array fabrication. (Step 1): Polyimide was formed on silicon (Si) substrates. (Step 2): A solution of indium(III) nitrate was spin-coated and thermally processed to form a thin layer of In_2O_3. (Step 3): The In_2O_3 layer was patterned *via* photolithography and dry etching. (Step 4): Electron-beam metal evaporation was used to produce Au/Ti source and drain electrodes patterned *via* photolithography. (Step 5): Polyimide layers were delaminated from Si substrates to obtain flexible sensor arrays.

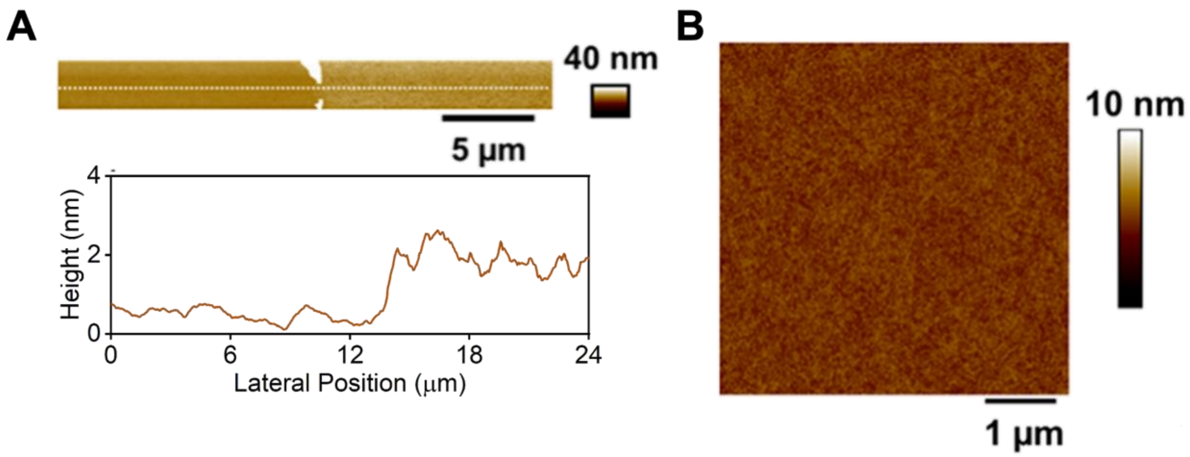

figure S2. Atomic force microscopy characterization of In_2O_3 thin films. (**A**) Atomic force microscope images of an In_2O_3 thin-film (~2-3 nm). The top image shows a step created by photolithography and dry etching as described in Methods, where polyimide/Si is on the left and In_2O_3/polyimide/Si is on the right. The bottom image shows the height profile along the dotted line shown in the top image. (**B**) Uniform deposition of In_2O_3 over a large surface area (root-mean-square roughness 0.34 nm).

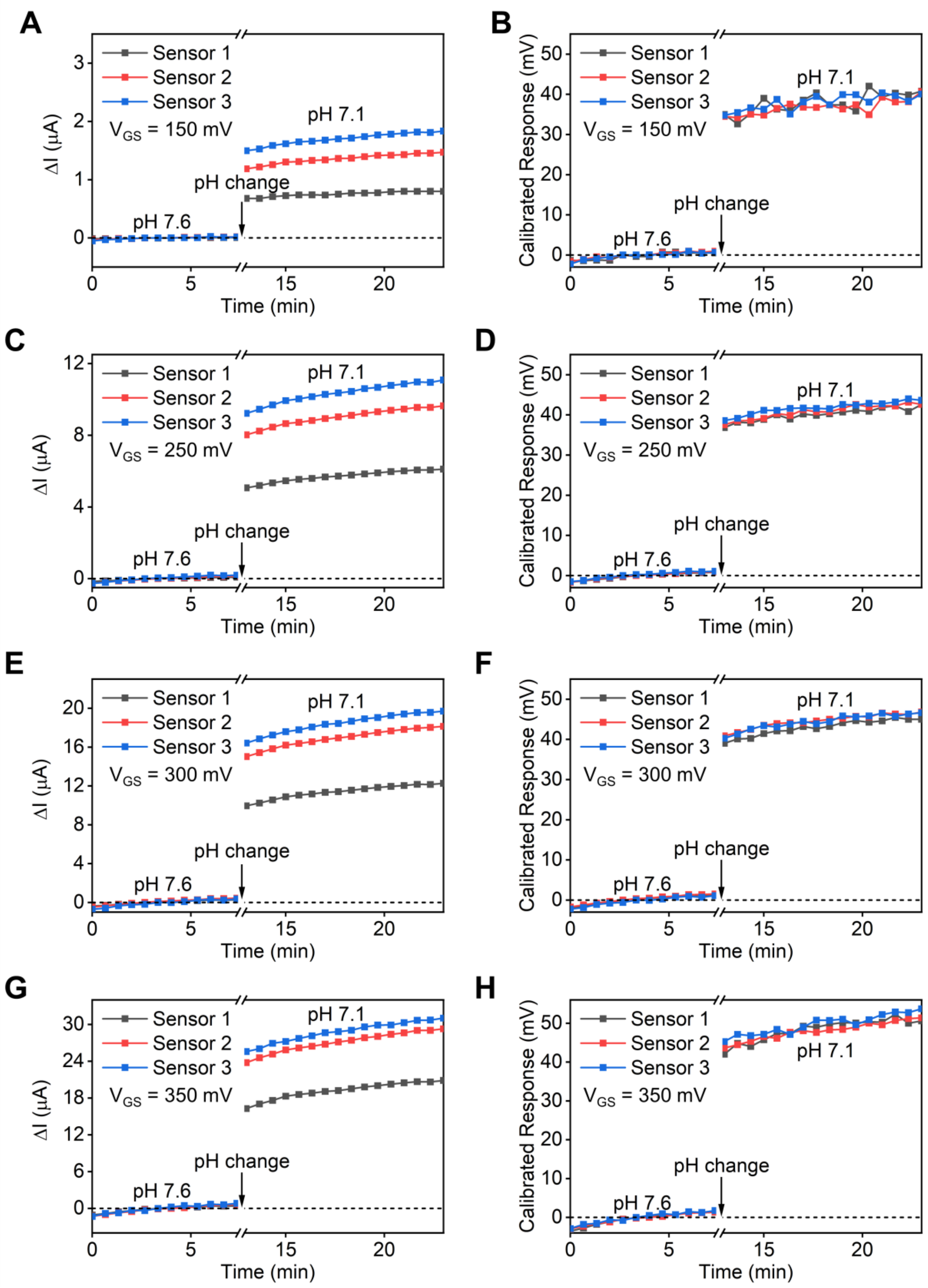

figure S3. Calibrated responses for field-effect transistor (FET)-based pH sensors at different gate voltage values (V_{GS}). (**A**,**C**,**E**,**G**) Real-time source-drain current (I_{DS}) changes (ΔI) for FET-based pH sensors associated with decreasing solution pH from 7.6 (baseline) to 7.1. V_{GS}=150, 250, 300, or 350 mV. (**B**,**D**,**F**,**H**) Corresponding calibrated responses for the FET-based pH sensors (V_{GS}=150, 250, 300, or 350 mV).

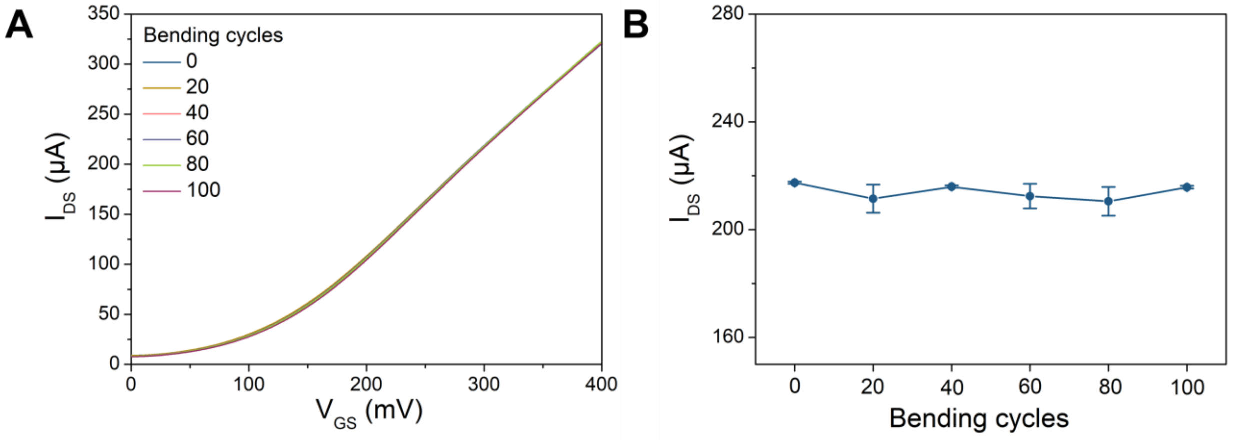

figure S4. Electronic stability of flexible In_2O_3 FETs after repeated bending cycles. (**A**) Transfer characteristics of a representative flexible In_2O_3 transistor before bending and after bending 20, 40, 60, 80, or 100 times. The bending radius was ∼15 mm. (**B**) Corresponding source-drain currents (I_{DS}) of the flexible In_2O_3 transistor (plotted at V_{GS} = 300 mV) after the different numbers of bending cycles shown in fig. S4A. Error bars are standard errors of the means for N = 3 repeated measurements.

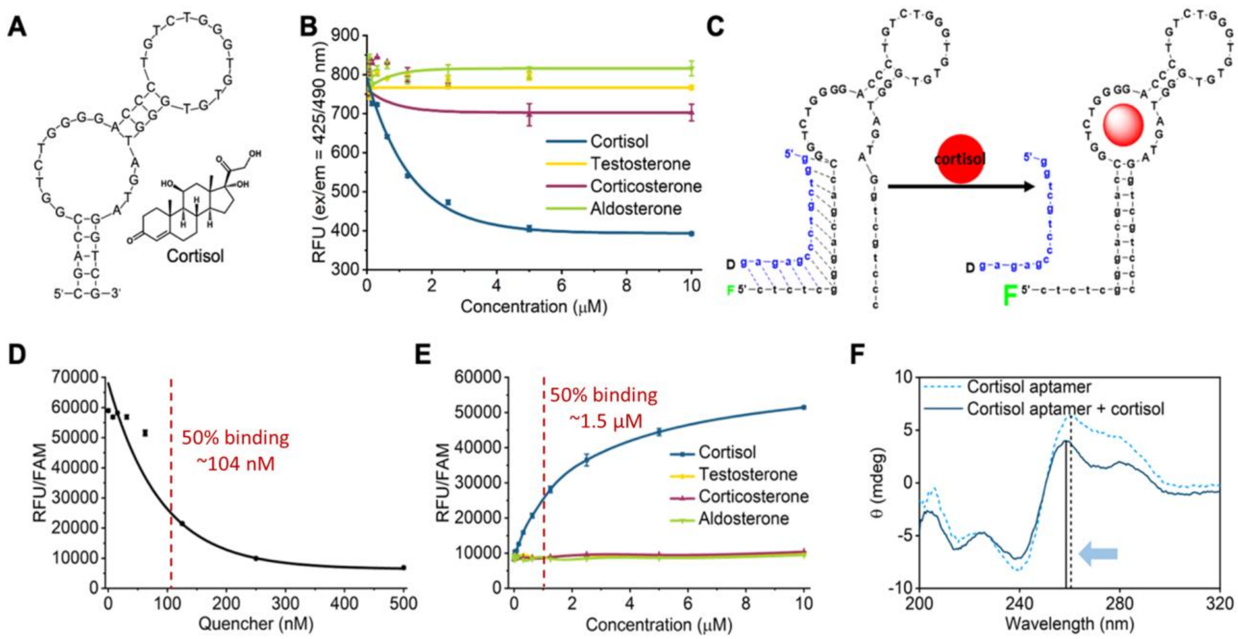

figure S5. Cortisol aptamer characterization. (**A**) Sequence and predicted secondary structure (*via mfold*) for the cortisol-specific aptamer. The inset shows the chemical structure of cortisol. (**B**) Using the sequence in (A), we carried out thioflavin T (ThT) dye displacement to compare target (cortisol) *vs.* non-target (testosterone, corticosterone, aldosterone) binding to the cortisol aptamer. Cortisol displaced aptamer-bound ThT producing a decrease in ThT fluorescence while non-targets did not displace ThT over the concentration range tested. (**C**) The aptamer sensor format for the fluorescein amidite (F) sensor/dabcyl (D) quencher structure-switching assay. Under competitive conditions, aptamer-target binding causes the aptamer to be released from the quencher strand such that the magnitude of the fluorescence response increases with increasing target concentration. (**D**) The association of a fluorescein (FAM)-conjugated aptamer with the (dabcyl) DAB-conjugated quencher strand was first measured in the absence of target. (**E**) The fluorescence response of the aptamer to cortisol and non-targets (testosterone, corticosterone, aldosterone). The K_d was calculated based on the plots in (D) and (E) where: $K_{d,eff1}$=[free aptamer][free quencher]/[aptamer-quencher]=79 nM, $K_{d,eff2}$=[free quencher][aptamer-target]/[aptamer-quencher][target]=0.1525 (unitless constant), and $K_d=K_{d,eff1}/K_{d,eff2}$=79 nM/0.1525=500 nM. (**F**) Circular dichroism spectra of the cortisol aptamer in artificial sweat before and after incubation with cortisol.

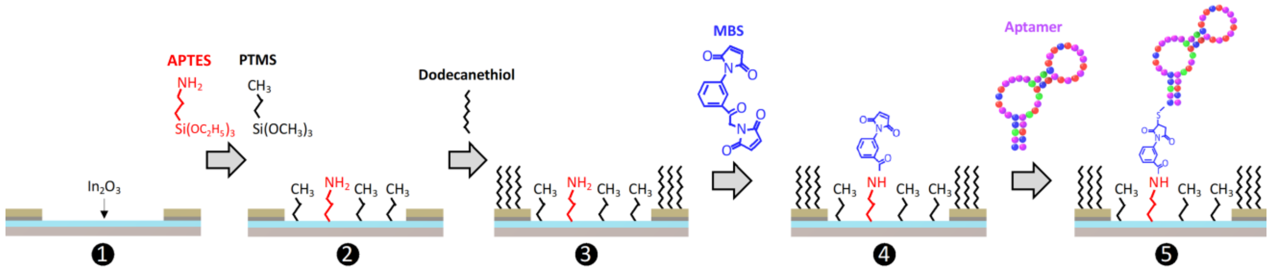

figure S6. Schematic of aptamer-field-effect transistor surface functionalization. The In_2O_3 channel (step 1) was silanized with (3-aminopropyl)triethoxysilane (APTES) and trimethoxy(propyl)silane (PTMS) (1:9) *via* self-assembly (step 2). The Au/Ti source and drain electrodes were passivated *via* self-assembly of 1-dodecanethiol monolayers (step 3). Amine-terminated silane molecules were reacted with 3-maleimidobenzoic acid *N*-hydroxysuccinimide ester (MBS) (step 4) to immobilize the thiolated cortisol aptamer (step 5).

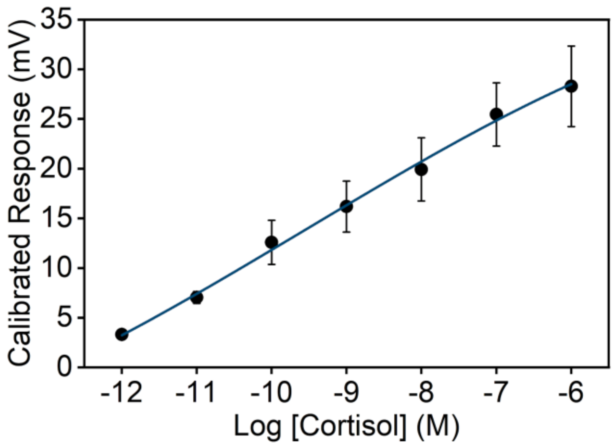

figure S7. Cortisol-aptamer-field-effect transistor (FET) calibrated responses in artificial saliva. Cortisol in artificial saliva was added to polydimethylsiloxane wells above FETs in increasing concentrations (1 pM to 1 μM). Error bars are standard errors of the means for determinations from *N*=3 FETs.

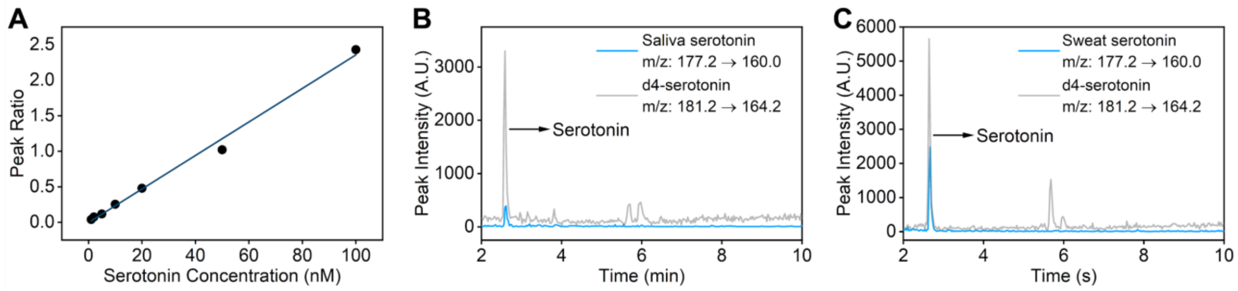

figure S8. Identification and characterization of serotonin in human saliva and sweat samples by liquid chromatography tandem mass spectrometry (LC-MS/MS). (**A**) A LC-MS/MS-based serotonin calibration plot. (**B**) Ion chromatograms of serotonin in a diluted human saliva sample. (**C**) Ion chromatograms of serotonin in a diluted human sweat sample.

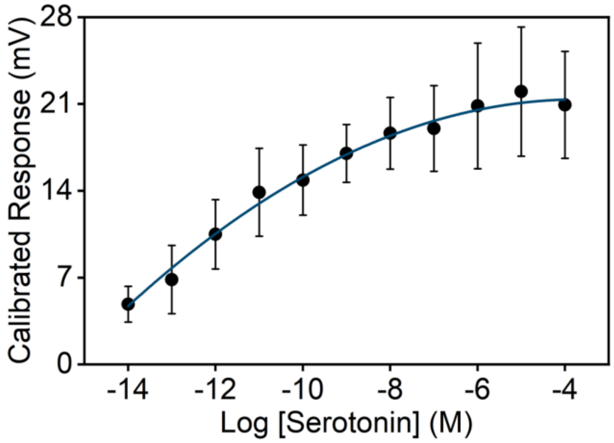

figure S9. Serotonin-aptamer-field-effect transistor (FET) calibrated responses in artificial sweat. Serotonin in artificial sweat was added to polydimethylsiloxane wells above FETs in increasing concentrations (10 fM to 100 μM). Error bars are standard errors of the means for determinations from *N*=3 FETs.

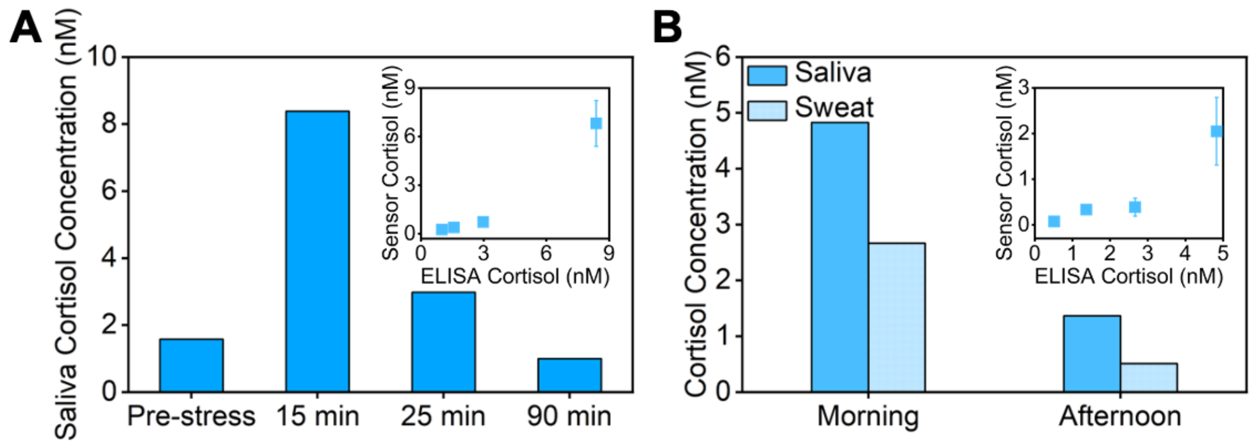

figure S10. Correlations between salivary or sweat cortisol levels determined by enzyme-linked immunosorbent assay (ELISA) *vs.* field-effect transistor (FET) measurements. (**A**) Salivary cortisol levels from a representative Trier Social Stress Test participant analyzed by ELISA. Inset: correlation between cortisol levels measured by a FET sensor (data shown in Fig. 3H) (r=0.98, $P<0.05$). (**B**) Morning and afternoon saliva and sweat cortisol levels from a representative participant analyzed by ELISA. Inset: correlation with cortisol levels measured by a FET sensor (data shown in Fig. 3J) (r = 0.87, $P<0.07$).

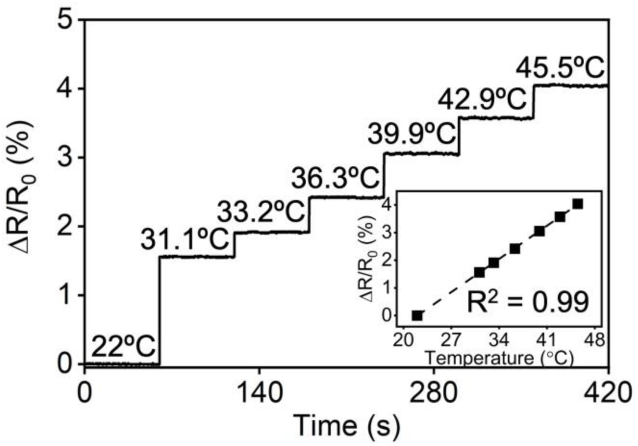

figure S11. Temperature sensor responses. The baseline resistance, R_0, at 22 °C (476 Ω), where ΔR corresponds to the change in resistance from baseline. The inset shows the corresponding temperature calibration plot.

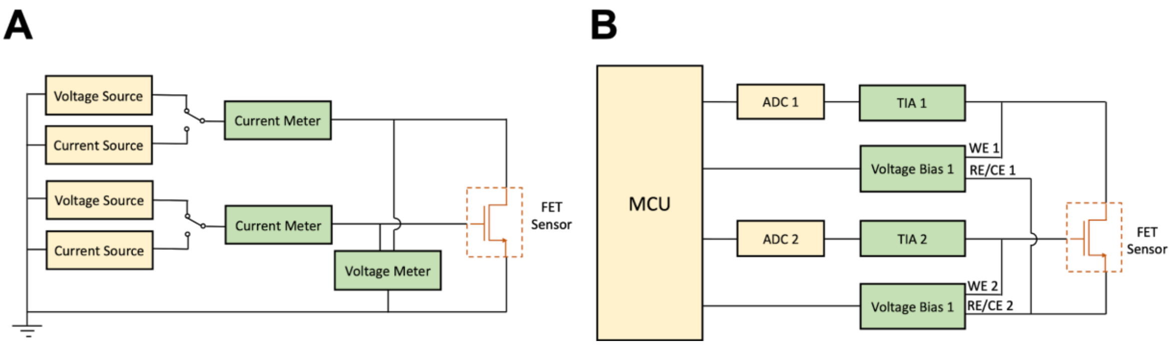

figure S12. Schematics of field-effect transistor (FET) signal acquisition (standard laboratory instrumentation). (**A**) A commercial source measurement unit (SMU). (**B**) A multi-channel potentiostat with a microcontroller unit (MCU) and two analog-digital converters (ADC) to control two 3-electrode configurations. Transimpedance amplifier (TIA), working electrode (WE), reference electrode (RE), and counter electrode (CE).

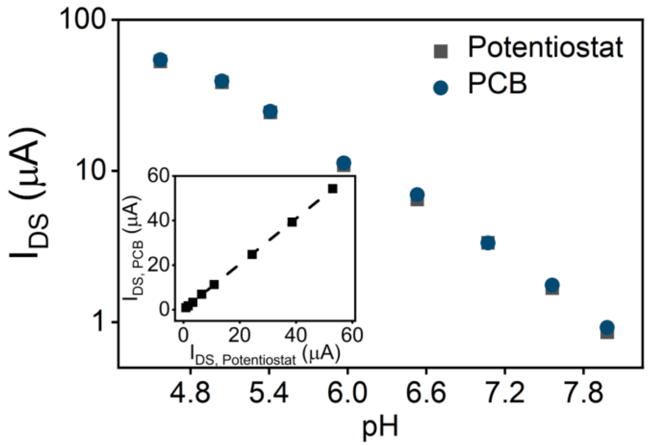

figure S13. Comparison of field-effect transistor source-drain current (I_{DS}) responses to pH changes from a custom-developed printed circuit board (PCB) *vs.* a multi-channel potentiostat. The inset shows the corresponding I_{DS} measurements made by the PCB *vs.* the standard laboratory instrument (*i.e.*, multi-channel potentiostat). All pH measurements were in phosphate-buffered saline.

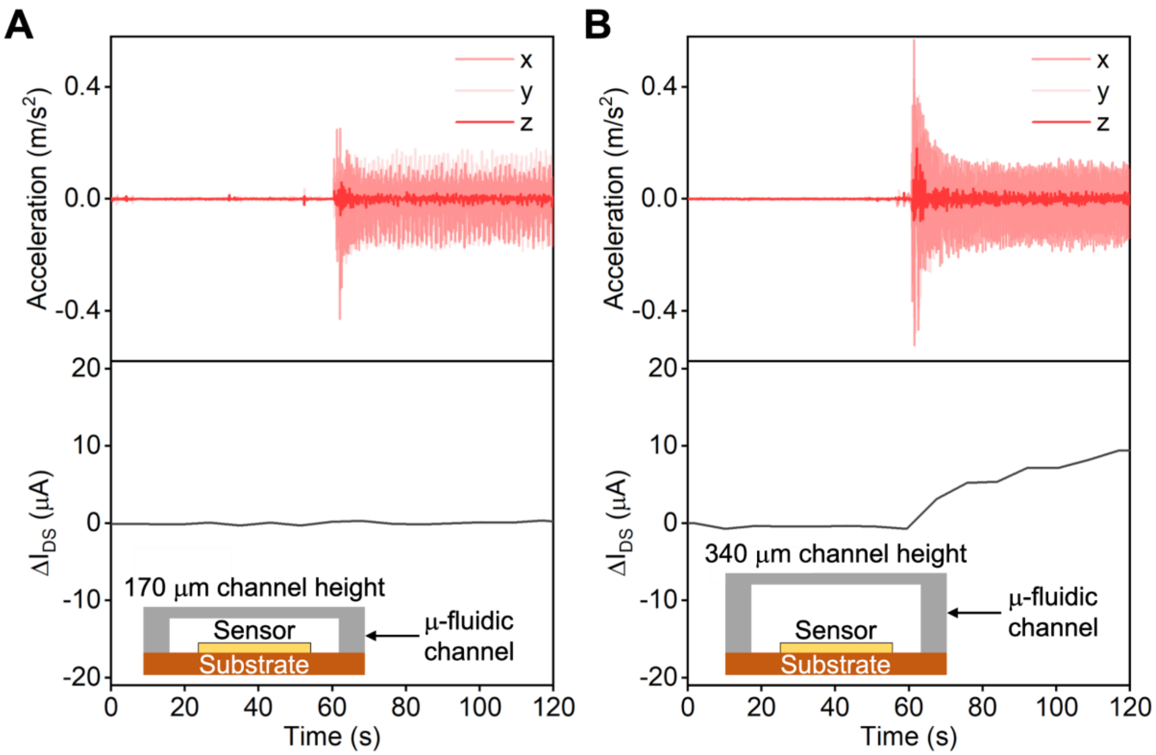

figure S14. *Ex-situ* effects of vertical vibration on field-effect transistor sensing responses for different microfluidic channel heights. Channel heights: (**A**) 170 μm, (**B**) 340 μm. The vibrational acceleration profiles are presented in the top half of each panel and the sensor responses (pH 7.5) are depicted on the bottom.

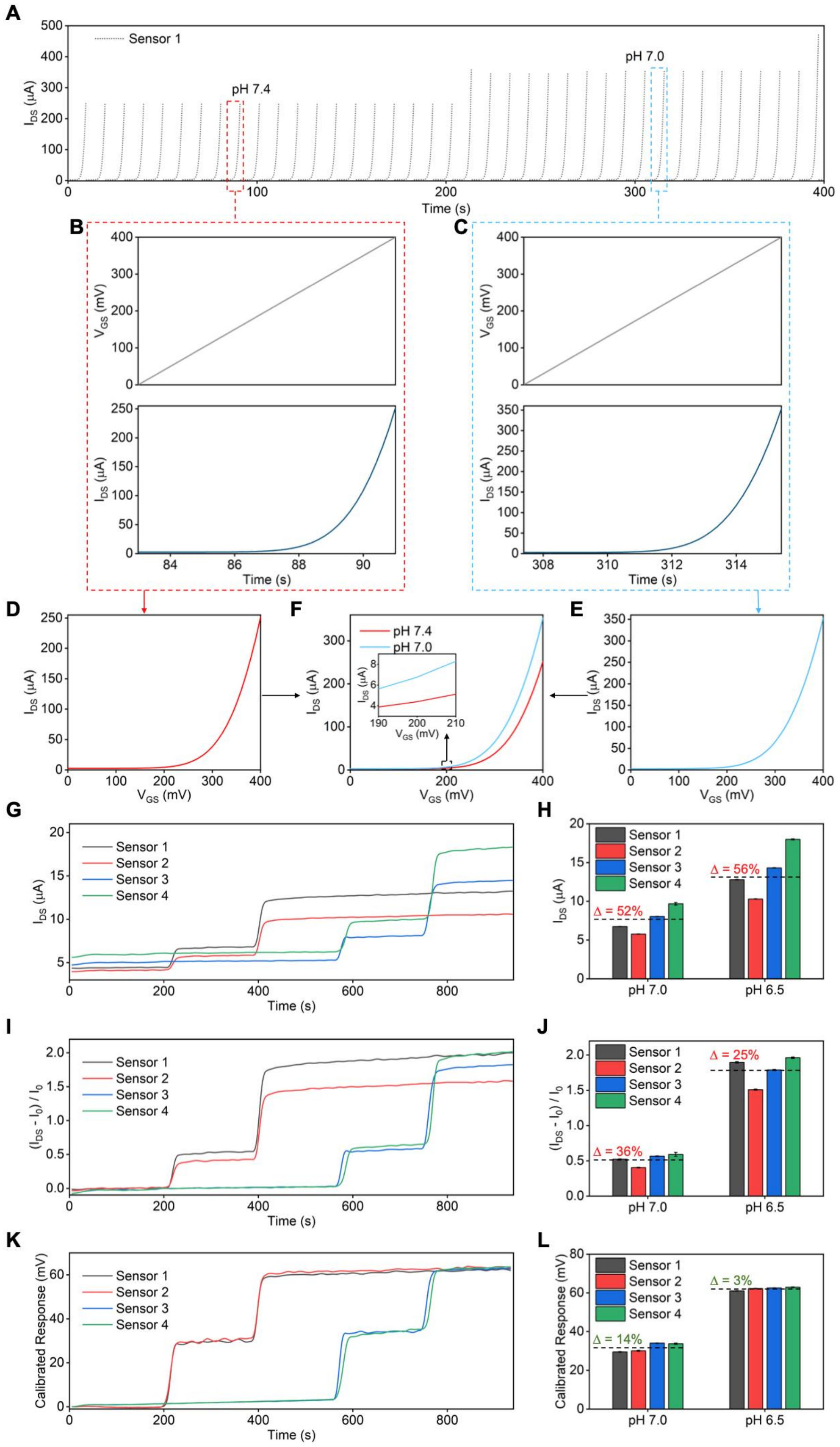

figure S15. Multi-channel pH data acquisition *via* a flexible printed circuit board. (**A**) Real-time recordings of source-drain current (I_{DS}) for a representative field-effect transistor (FET)-based pH sensor with gate voltage (V_{GS}) sweep to track dynamic variations in FET transfer curves in response to a change in pH from 7.4 to pH 7.0 in phosphate-buffered saline (PBS). (**B**, **C**) Representative V_{GS} scans (top) and I_{DS} recordings (bottom) at baseline (pH 7.4) and pH 7.0. (**D**, **E**) The transfer curves acquired for baseline (pH 7.4) and pH 7.0. (**F**) The overlaid transfer curves for the baseline (pH 7.4) and pH 7.0 measurements. The inset shows a high resolution view of the

overlaid transfer curves demonstrating that each curve is distinguishable. (**G**) Temporal I_{DS} monitoring to track FET responses to changes in pH from baseline (pH 7.4) to pH 7.0 (sensors 1,2 @200 s; sensors 3,4 @580 s) to pH 6.5 (sensors 1,2 @400 s; sensors 3,4 @780 s) in PBS. (**H**). Mean I_{DS} responses for four FETs showing large device-to-device variations (52% @pH 7.0; 56% @pH 6.5). (**I**) The same data shown in (G) normalized to I_{DS} at baseline (pH 7.4). (**J**). Mean normalized I_{DS} responses for the four FETs continue to show device-to-device variation (36% @pH 7.0; 25% @pH 6.5). (**K**) The same data in (G) depicted as calibrated responses with respect to time. (**L**) Mean calibrated responses for the four FETs show reduced/minimal device-to-device variation (14% @pH 7.0; 3% @pH 6.5).

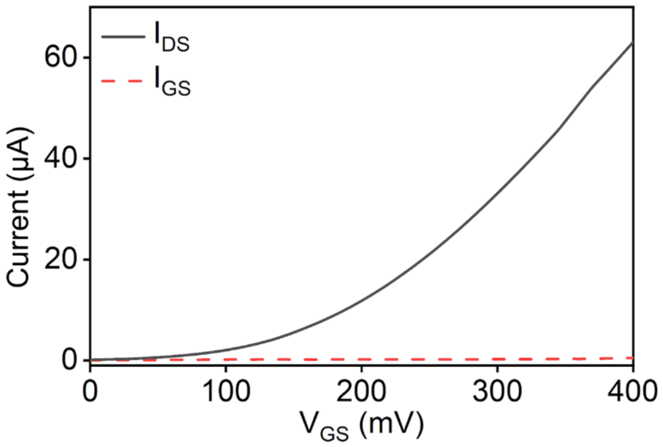

figure S16. Characterization of leakage current through a representative gate electrode. Minimal leakage current (I_{GS}) through a Ag/AgCl reference electrode was observed relative to the source-drain current (I_{DS}) in artificial sweat.

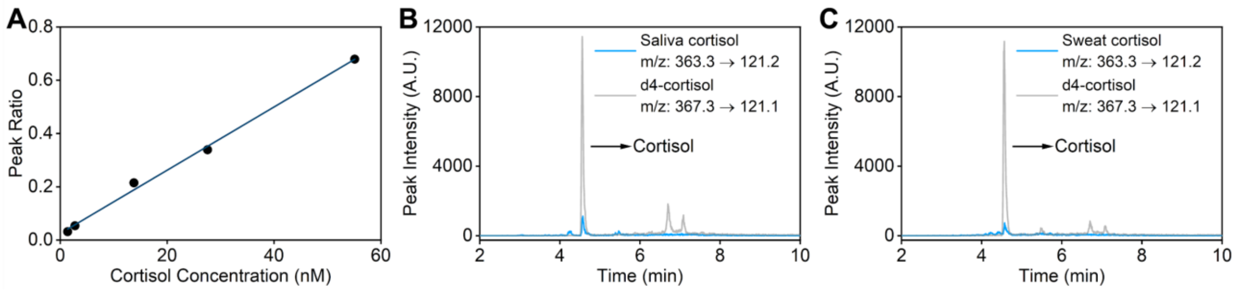

figure S17. Characterization of cortisol in human saliva and sweat samples by liquid chromatography tandem mass spectrometry(LC-MS/MS). (**A**) A LC-MS/MS-based cortisol calibration plot. (**B**) Ion chromatograms of cortisol in a diluted human saliva sample. (**C**) Ion chromatograms of cortisol in a diluted human sweat sample.

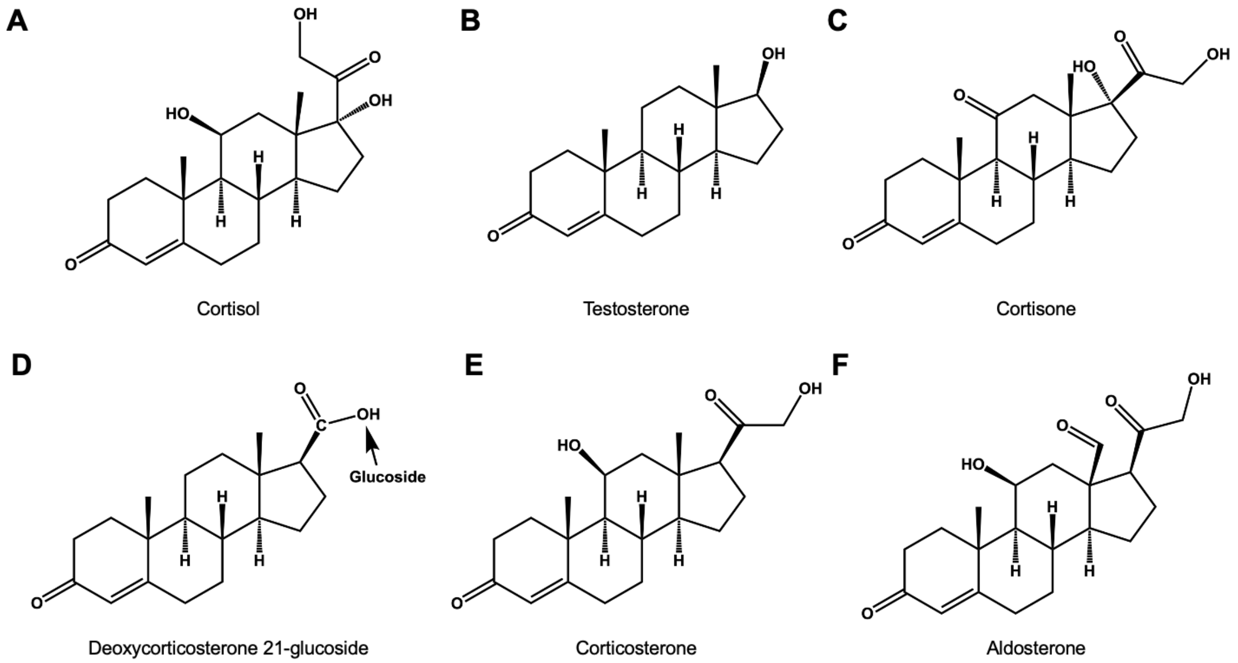

figure S18. Summary of chemical structures.

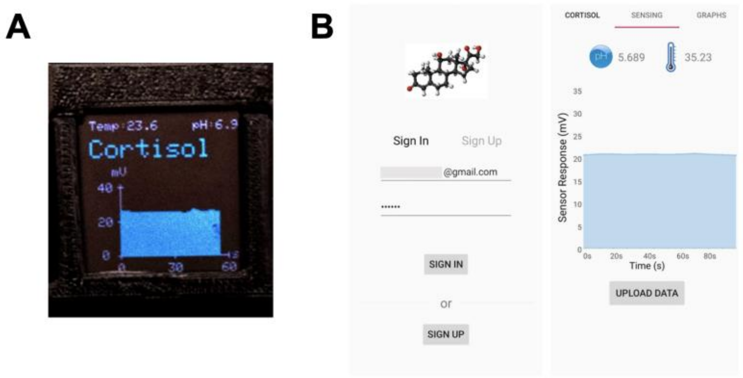

figure S19. Images for the aptamer-FET biosensing smartwatch and smartphone application. (A) Smartwatch screen. (B) Screenshots of the login and sensing pages of the smartphone Android application. Photo Credit: Zhaoqing Wang, Hannaneh Hojaiji, UCLA.

**Reference**	**Biorecognition element**	**Signal transduction method**	**Direct and label-free detection?**	**With an integrated wireless System?**	**Human subjects studied?**	**Detection limit or lowest conc. investigated**
This Work	Aptamer (previously unreported)	Field-effect transistor (FET)	Yes	Yes	Yes<br>88 subjects	0.001 nM
(*17*)	Molecularly imprinted polymer (MIP)	Organic electrochemical transistor (OECT)	Yes	No	Yes<br>2 subjects	0.1 nM
(*18*)	Antibody	Colorimetric (Lateral flow immunoassay)	No<br>•Multi-step operations and external reagents needed.	Not applicable	Yes<br>4 subjects	13.79 nM (5 ng/mL)
(*19*)	Antibody	Electrochemical (amperometry)	No<br>•Multi-step operations and external reagents needed.	Yes	Yes<br>12 subjects	0.221 nM (0.08 ng/mL)
(*20*)	Aptamer	Nonfaradaic electro-chemical impedence spectroscopy (EIS)	Yes	No	Yes<br>3 subjects	11.03 nM (4 ng/mL)
(21)	MIP	Electrochemical (amperometry)	No<br>•Assisted by redox probes (*i.e.*, Prussian blue)	Yes (touch-based portable system)	Yes<br>7 subjects	1 nM
(*22*)	Antibody	EIS	No<br>•External reagent delivery needed (*i.e.*, $K_3[Fe(CN)_6]$)	No	Yes<br>3 subjects	0.0028 nM (1 pg/mL)
(*23*)	Antibody	Electrochemical (differential pulse voltammetry)	No<br>•Multi-step operations and external reagents needed.	Yes	Yes<br>2 subjects	7.47 nM
(*24*)	Antibody	Electrochemical (amperometry)	No	Yes	Yes<br>1 subject	0.0028 nM (1 pg/mL)

(34)	Antibody	FET	Yes	No	No	2.76 nM (1 ng/mL)
(*35*)	Aptamer	Electrochemical (non-faradaic EIS)	Yes	No	Yes 5 subjects	2.76 nM (1 ng/mL)
(*81*)	Aptamer	Graphene FET	Yes	Yes	Yes 1 subject	0.01 nM
(*82*)	Antibody	Graphene FET	Yes	Yes	Yes 1 subject	0.028 nM (10 pg/ml)
(*83*)	Aptamer	Electrochemical (differential pulse voltammetry)	No •Redox probe assisted	No	No	1 nM

table S1. Comparison of recent noninvasive cortisol sensing platforms.

Thiolated cortisol aptamer sequence	5'-/5ThioMC6-D/CGACCGGTCTGGGGACCCTGTCTGGGTGTGTGGGTAGTAGGTCG-3'
Cortisol aptamer sequence with fluorescein at the 5'-end	5'-/56-FAM/CTC TCG GGA CGA CCG GTC TGG GGA CCC TGT CTG GGT GTG TGG GTA GTA GGT CGT CCC-3'
Quencher strand with dabcyl at the 3'-end	5'- GGT CGT CCC GAG AG/3Dab/-3'
Scrambled cortisol aptamer sequence	5'-/5ThioMC6-D/CCACCGCAGTCCGGTCGCTTGCTCGCTGTGTGGGTAGTAGGTCG-3'
Thiolated serotonin aptamer sequence	5'-/5ThioMC6-D/CGACTGGTAGGCAGATAGGGGAAGCTGATTCGATGCGTGGGTCG-3'
Oligonucleotides used in selection process	
N36 random library	5'-GGA GGC TCT CGG GAC GAC- (N_{36})-GTC GTC CCG CCT TTA GGA TTT ACA G-3'
Biotinylated column immobilizing capture strand	5'-GTC GTC CCG AGA GCC ATA/3BioTEG/
Forward PCR primer	5'-GGA GGC TCT CGG GAC GAC-3'
Reverse PCR primer	5'-CTG TAA ATC CTA AAG GCG GGA CGA C-3'
Biotinylated PCR reverse-primer	5'-/5Biosg/ CTG TAA ATC CTA AAG GCG GGA CGA C-3'

table S2. Oligonucleotide sequences.

Rounds	Target	Counter Target	Buffer Condition
1	100 µM, #3	DOG, 5 µM #16	PBS + 2 mM $MgCl_2$
2	50 µM, #3	DOG, 5 µM #16	PBS + 2 mM $MgCl_2$
3	40 µM, #3	DOG, 5 µM #16	PBS + 2 mM $MgCl_2$
4-5	30 µM, #3	TES, 5 µM #16	PBS + 2 mM $MgCl_2$
6	20 µM, #3	The mixture of DOG/TES, each 5 µM, #16	PBS + 2 mM $MgCl_2$
7	10 µM, #3	No counter target	NxStage pureflow
8	5 µM, #3	Mixture of DOG/TES, each 5 µM, #20	NxStage pureflow
9	3 µM, #3	Mixture of DOG/TES, each 5 µM, #20	NxStage pureflow
10	3 µM, #3	Aldosterone, 3 µM, #16	PBS + 2 mM $MgCl_2$
11	2 µM, #3	Mixture of TES/cortisone, each 2 µM, #20	PBS + 2 mM $MgCl_2$
12	2 µM, #3	DOG,2 µM, #20	PBS + 2 mM $MgCl_2$
13	1 µM, #3	TES, 2 µM, #24	PBS + 2 mM $MgCl_2$
14	1 µM, #3	Cortisone, 1 µM, #24	PBS + 2 mM $MgCl_2$
15	0.5 µM, #3	Cortisone, 2 µM, #20	PBS + 2 mM $MgCl_2$
16	0.5 µM, #3	Cortisone, 0.5 µM, #20	PBS + 2 mM $MgCl_2$
17	0.4 µM, #3	No counter target	NxStage pureflow
18-19	0.2 µM, #3	No counter target	PBS + 2 mM $MgCl_2$

table S3. Cortisol aptamer selection process. The numbers of rounds of selection and counter-selection were determined empirically using the polymerase chain reaction product elution profiles on agarose gels. Sufficient selection was determined to have occurred when significantly brighter PCR bands were observed from target elution steps compared to the last wash and the "counter-target" elutions. The '#' indicate the number of elutions. An artificial dialysis solution (NxStage pureflow) was used where indicated during selection to isolate a cortisol aptamer for kidney dialysis applications, as well as for use in the physiological fluid sensing applications herein. We carried out new selections because our previously reported cortisol aptamers *(41)* were isolated under high salt conditions (20 mM HEPES, 1 M NaCl, 10 mM $MgCl_2$, 5 mM KCl, pH 7.5). As such, the previous aptamers showed inadequate target recognition under physiological salt conditions (*e.g.*, in saliva and sweat). The ionic compositions, including NaCl concentrations, of the artificial sweat and artificial saliva solutions used in the experiments herein are shown in table S4. The target is cortisol. Deoxycorticosterone 21-glucoside, DOG; testosterone, TES; phosphate-buffered saline, PBS. Chemical structures are shown in fig. S18.

**Group**	**Sub group**	**Sequence Alignment (5' → 3')**			**Count**
		Conserved region	Random region	Conserved region	
1		ctctcgggacgac	**CGCCAGAAAGAA-----TGAGGATAGGC-TAGGATAGCCTAG**	gtcgtccc	2
		ctctcgggacgac	**CGCCAGAAACTTG----TGAGGATAGGTGTAGCA---CCTAG**	gtcgtccc	10
		ctctcgggacgac	**CGCCAGAA-GATCGCATCGAGGATAGTTCACAA-----CTAG**	gtcgtccc	7
2	a	ctctcgggacgac	**TACA-TGGGTGTGTGGGTAGGTCTGGGGACCCGGTG**	gtcgtccc	2
		ctctcgggacgac	**CATGTTGGGTGTGTGGGTAGGTCTGGGGACCCGGTG**	gtcgtccc	1
	b	ctctcgggacgac	**CGGTCTGGGGACCCTGTCTGGGTGTGTGGGTAGTAG**	gtcgtccc	1

table S4. Cloned sequences from the cortisol aptamer selections. Twenty-three clones were sequenced. Copy number redundancies and sequence homologies were analyzed (Multali, http://multalin.toulouse.inra.fr/multalin/multalin.html). Lower case letters represent conserved sequence regions. Capital letters indicate the random region. Colored letters show common motifs. Aptamer 2b was used in this study.

Artificial Sweat (pH=7)	
**Ion**	**Concentration (mM)**
Na^{+}	33.1
Zn^{2+}	0.0112
Ca^{2+}	1.00
Fe^{3+}	0.0046
Mg^{2+}	0.0553
K^{+}	6.99
Cl^{-}	42.1
SO_4^{2-}	0.069

Artificial Saliva (pH=6.8)	
**Ion**	**Concentration (mM)**
Na^{+}	5.65
Ca^{2+}	1.02
Mg^{2+}	0.836
K^{+}	10.1
Cl^{-}	19.4
HPO_4^{2-}	3.33
CO_3^{2-}	3.84

table S5. Buffer compositions. Ionic compositions of artificial sweat (I2BL-0011, Pickering Laboratories, Inc., Mountain View, CA) and artificial saliva (1700-0303, Pickering Laboratories).

REFERENCES AND NOTES

1. J. Heikenfeld, A. Jajack, B. Feldman, S. W. Granger, S. Gaitonde, G. Begtrup, B. A. Katchman, Accessing analytes in biofluids for peripheral biochemical monitoring. *Nat. Biotechnol.* **37**, 407–419 (2019).

2. T. R. Ray, J. Choi, A. J. Bandodkar, S. Krishnan, P. Gutruf, L. Tian, R. Ghaffari, J. A. Rogers, Bio-integrated wearable systems: A comprehensive review. *Chem. Rev.* **119**, 5461–5533 (2019).

3. X. Cheng, B. Wang, Y. Zhao, H. Hojaiji, S. Lin, R. Shih, H. Lin, S. Tamayosa, B. Ham, P. Stout, K. Salahi, Z. Wang, C. Zhao, J. Tan, S. Emaminejad, A mediator-free electroenzymatic sensing methodology to mitigate ionic and electroactive interferents' effects for reliable wearable metabolite and nutrient monitoring. *Adv. Funct. Mater.* **30**, 1908507 (2020).

4. W. Gao, S. Emaminejad, H. Y. Y. Nyein, S. Challa, K. Chen, A. Peck, H. M. Fahad, H. Ota, H. Shiraki, D. Kiriya, D.-H. Lien, G. A. Brooks, R. W. Davis, A. Javey, Fully integrated wearable sensor arrays for multiplexed in situ perspiration analysis. *Nature* **529**, 509–514 (2016).

5. J. Kim, A. S. Campbell, B. E.-F. de Ávila, J. Wang, Wearable biosensors for healthcare monitoring. *Nat. Biotechnol.* **37**, 389–406 (2019).

6. Y. Zhao, B. Wang, H. Hojaiji, Z. Wang, S. Lin, C. Yeung, H. Lin, P. Nguyen, K. Chiu, K. Salahi, X. Cheng, J. Tan, B. A. Cerrillos, S. Emaminejad, A wearable freestanding electrochemical sensing system. *Sci. Adv.* **6**, eaaz0007 (2020).

7. A. Clow, F. Hucklebridge, T. Stalder, P. Evans, L. Thorn, The cortisol awakening response: More than a measure of HPA axis function. *Neurosci. Biobehav. Rev.* **35**, 97–103 (2010).

8. E. K. Adam, S. Vrshek-Schallhorn, A. D. Kendall, S. Mineka, R. E. Zinbarg, M. G. Craske, Prospective associations between the cortisol awakening response and first onsets of anxiety disorders over a six-year follow-up–2013 Curt Richter Award Winner. *Psychoneuroendocrinology* **44**, 47–59 (2014).

9. P. Restituto, J. Galofré, M. Gil, C. Mugueta, S. Santos, J. Monreal, N. Varo, Advantage of salivary cortisol measurements in the diagnosis of glucocorticoid related disorders. *Clin. Biochem.* **41**, 688–692 (2008).

10. R. Yehuda, M. H. Teicher, R. L. Trestman, R. A. Levengood, L. J. Siever, Cortisol regulation in posttraumatic stress disorder and major depression: A chronobiological analysis. *Biol. Psychiatry* **40**, 79–88 (1996).

11. A. C. Incollingo Rodriguez, E. S. Epel, M. L. White, E. C. Standen, J. R. Seckl, A. J. Tomiyama, Hypothalamic-pituitary-adrenal axis dysregulation and cortisol activity in obesity: A systematic review. *Psychoneuroendocrinology* **62**, 301–318 (2015).

12. M. Akinola, E. Page-Gould, P. H. Mehta, J. G. Lu, Collective hormonal profiles predict group performance. *Proc. Natl. Acad. Sci. U.S.A.* **113**, 9774–9779 (2016).

13. S. Hart, L. M. Boylan, B. Border, S. R. Carroll, D. McGunegle, R. M. Lampe, Breast milk levels of cortisol and secretory immunoglobulin A (SIgA) differ with maternal mood and infant neuro-behavioral functioning. *Infant Behav. Dev.* **27**, 101–106 (2004).

14. U. Teruhisa, H. Ryoji, I. Taisuke, S. Tatsuya, M. Fumihiro, S. Tatsuo, Use of saliva for monitoring unbound free cortisol levels in serum. *Clin. Chim. Acta* **110**, 245–253 (1981).

15. R. F. Vining, R. A. McGinley, J. J. Maksvytis, K. Y. Ho, Salivary cortisol: A better measure of adrenal cortical function than serum cortisol. *Ann. Clin. Biochem.* **20**, 329–335 (1983).

16. J. Heikenfeld, Non-invasive analyte access and sensing through eccrine sweat: Challenges and outlook circa 2016. *Electroanalysis* **28**, 1242–1249 (2016).

17. O. Parlak, S. T. Keene, A. Marais, V. F. Curto, A. Salleo, Molecularly selective nanoporous membrane-based wearable organic electrochemical device for noninvasive cortisol sensing. *Sci. Adv.* **4**, eaar2904 (2018).

18. S. Kim, B. Lee, J. T. Reeder, S. H. Seo, S.-U. Lee, A. Hourlier-Fargette, J. Shin, Y. Sekine, H. Jeong, Y. S. Oh, A. J. Aranyosi, S. P. Lee, J. B. Model, G. Lee, M.-H. Seo, S. S. Kwak, S. Jo, G. Park, S. Han, I. Park, H.-I. Jung, R. Ghaffari, J. Koo, P. V. Braun, J. A. Rogers, Soft, skin-interfaced microfluidic systems with integrated immunoassays, fluorometric sensors, and impedance measurement capabilities. *Proc. Natl. Acad. Sci. U.S.A.* **117**, 27906–27915 (2020).

19. R. M. Torrente-Rodríguez, J. Tu, Y. Yang, J. Min, M. Wang, Y. Song, Y. Yu, C. Xu, C. Ye, W. W. IsHak, W. Gao, Investigation of cortisol dynamics in human sweat using a graphene-based wireless mHealth system. *Matter* **2**, 921–937 (2020).

20. A. Ganguly, K. C. Lin, S. Muthukumar, S. Prasad, Autonomous, real-time monitoring electrochemical aptasensor for circadian tracking of cortisol hormone in sub-microliter volumes of passively eluted human sweat. *ACS Sens.* **6**, 63–72 (2021).

21. W. Tang, L. Yin, J. R. Sempionatto, J. M. Moon, H. Teymourian, J. Wang, Touch-based stressless cortisol sensing. *Adv. Mater.* **33**, 2008465 (2021).

22. H.-B. Lee, M. Meeseepong, T. Q. Trung, B.-Y. Kim, N.-E. Lee, A wearable lab-on-a-patch platform with stretchable nanostructured biosensor for non-invasive immunodetection of biomarker in sweat. *Biosens. Bioelectron.* **156**, 112133 (2020).

23. C. Cheng, X. Li, G. Xu, Y. Lu, S. S. Low, G. Liu, L. Zhu, C. Li, Q. Liu, Battery-free, wireless, and flexible electrochemical patch for in situ analysis of sweat cortisol via near field communication. *Biosens. Bioelectron.* **172**, 112782 (2021).

24. P. Rice, S. Upasham, B. Jagannath, R. Manuel, M. Pali, S. Prasad, CortiWatch: Watch-based cortisol tracker. *Future Sci. OA* **5**, FSO416 (2019).

25. Y. Xiao, A. A. Lubin, A. J. Heeger, K. W. Plaxco, Label-free electronic detection of thrombin in blood serum by using an aptamer-based sensor. *Angew. Chem. Int. Ed.* **117**, 5592–5595 (2005).

26. H. Li, P. Dauphin-Ducharme, G. Ortega, K. W. Plaxco, Calibration-free electrochemical biosensors supporting accurate molecular measurements directly in undiluted whole blood. *J. Am. Chem. Soc.* **139**, 11207–11213 (2017).

27. K. M. Cheung, K.-A. Yang, N. Nakatsuka, C. Zhao, M. Ye, M. E. Jung, H. Yang, P. S. Weiss, M. N. Stojanović, A. M. Andrews, Phenylalanine monitoring via aptamer-field-effect transistor sensors. *ACS Sens.* **4**, 3308–3317 (2019).

28. N. Nakatsuka, K.-A. Yang, J. M. Abendroth, K. M. Cheung, X. Xu, H. Yang, C. Zhao, B. Zhu, Y. S. Rim, Y. Yang, P. S. Weiss, M. N. Stojanović, A. M. Andrews, Aptamer-field-effect transistors overcome Debye length limitations for small-molecule sensing. *Science* **362**, 319–324 (2018).

29. C. Zhao, Q. Liu, K. M. Cheung, W. Liu, Q. Yang, X. Xu, T. Man, P. S. Weiss, C. Zhou, A. M. Andrews, Narrower nanoribbon biosensors fabricated by chemical lift-off lithography show higher sensitivity. *ACS Nano* **15**, 904–915 (2021).

30. Q. Liu, C. Zhao, M. Chen, Y. Liu, Z. Zhao, F. Wu, Z. Li, P. S. Weiss, A. M. Andrews, C. Zhou, Flexible multiplexed In_2O_3 nanoribbon aptamer-field-effect transistors for biosensing. *iScience* **23**, 101469 (2020).

31. C. Zhao, K. M. Cheung, I.-W. Huang, H. Yang, N. Nakatsuka, W. Liu, Y. Cao, T. Man, P. S. Weiss, H. G. Monbouquette, A. M. Andrews, Implantable aptamer–field-effect transistor neuroprobes for in vivo neurotransmitter monitoring. *Sci. Adv.* **7**, eabj7422 (2021).

32. Y. S. Rim, S.-H. Bae, H. Chen, J. L. Yang, J. Kim, A. M. Andrews, P. S. Weiss, Y. Yang, H.-R. Tseng, Printable ultrathin metal oxide semiconductor-based conformal biosensors. *ACS Nano* **9**, 12174–12181 (2015).

33. F. N. Ishikawa, M. Curreli, H.-K. Chang, P.-C. Chen, R. Zhang, R. J. Cote, M. E. Thompson, C. Zhou, A calibration method for nanowire biosensors to suppress device-to-device variation. *ACS Nano* **3**, 3969–3976 (2009).

34. H.-J. Jang, T. Lee, J. Song, L. Russell, H. Li, J. Dailey, P. C. Searson, H. E. Katz, Electronic cortisol detection using an antibody-embedded polymer coupled to a field-effect transistor. *ACS Appl. Mater. Interfaces* **10**, 16233–16237 (2018).

35. M. Pali, B. Jagannath, K.-C. Lin, S. Upasham, D. Sankhalab, S. Upashama, S. Muthukumar, S. Prasad, CATCH (Cortisol Apta WATCH): 'Bio-mimic alarm' to track anxiety, stress, immunity in human sweat. *Electrochim. Acta* **390**, 138834 (2021).

36. J. Kim, Y. S. Rim, H. Chen, H. H. Cao, N. Nakatsuka, H. L. Hinton, C. Zhao, A. M. Andrews, Y. Yang, P. S. Weiss, Fabrication of high-performance ultrathin In_2O_3 film field-effect transistors and biosensors using chemical lift-off lithography. *ACS Nano* **9**, 4572–4582 (2015).

37. H. Chen, Y. S. Rim, I. C. Wang, C. Li, B. Zhu, M. Sun, M. S. Goorsky, X. He, Y. Yang, Quasi-two-dimensional metal oxide semiconductors based ultrasensitive potentiometric biosensors. *ACS Nano* **11**, 4710–4718 (2017).

38. Y. S. Rim, H. Chen, T.-B. Song, S.-H. Bae, Y. Yang, Hexaaqua metal complexes for low-temperature formation of fully metal oxide thin-film transistors. *Chem. Mater.* **27**, 5808–5812 (2015).

39. P. S. Weiss, P. L. Trevor, M. J. Cardillo, Gas–surface interactions on InP monitored by changes in substrate electronic properties. *J. Chem. Phys.* **90**, 5146–5153 (1989).

40. A. Many, Y. Goldstein, N. B. Grover, *Semiconductor Surfaces* (North-Holland Publishing Co., Amsterdam, 1965).

41. K.-A. Yang, H. Chun, Y. Zhang, S. Pecic, N. Nakatsuka, A. M. Andrews, T. S. Worgall, M. N. Stojanović, High-affinity nucleic-acid-based receptors for steroids. *ACS Chem. Biol.* **12**, 3103–3112 (2017).

42. K.-A. Yang, R. Pei, M. N. Stojanović, In vitro selection and amplification protocols for isolation of aptameric sensors for small molecules. *Methods* **106**, 58–65 (2016).

43. N. Nakatsuka, J. M. Abendroth, K. A. Yang, A. M. Andrews, Divalent cation dependence enhances dopamine aptamer biosensing. *ACS Appl. Mater. Interfaces* **13**, 9425–9435 (2021).

44. J. Kypr, I. Kejnovská, D. Renčiuk, M. Vorlíčková, Circular dichroism and conformational polymorphism of DNA. *Nucleic Acids Res.* **37**, 1713–1725 (2009).

45. O. Neumann, D. Zhang, F. Tam, S. Lal, P. Wittung-Stafshede, N. J. Halas, Direct optical detection of aptamer conformational changes induced by target molecules. *Anal. Chem.* **81**, 10002–10006 (2009).

46. V. B. Juska, M. E. Pemble, A critical review of electrochemical glucose sensing: Evolution of biosensor platforms based on advanced nanosystems. *Sensors* **20**, 6013 (2020).

47. M. Trilck, J. Flitsch, D. Lüdecke, R. Jung, S. Petersenn, Salivary cortisol measurement-a reliable method for the diagnosis of Cushing's syndrome. *Exp. Clin. Endocrinol. Diabetes* **113**, 225–230 (2005).

48. R. Miller, F. Plessow, M. Rauh, M. Gröschl, C. Kirschbaum, Comparison of salivary cortisol as measured by different immunoassays and tandem mass spectrometry. *Psychoneuroendocrinology* **38**, 50–57 (2013).

49. M. Jia, W. M. Chew, Y. Feinstein, P. Skeath, E. M. Sternberg, Quantification of cortisol in human eccrine sweat by liquid chromatography–tandem mass spectrometry. *Analyst* **141**, 2053–2060 (2016).

50. C. Muir, K. Treasurywala, S. McAllister, J. Sutherland, L. Dukas, R. Berger, A. Khan, D. DeCatanzaro, Enzyme immunoassay of testosterone, 17β-estradiol, and progesterone in

perspiration and urine of preadolescents and young adults: Exceptional levels in men's axillary perspiration. *Horm. Metab. Res.* **40**, 819–826 (2008).

51. K. Ngamchuea, K. Chaisiwamongkhol, C. Batchelor-McAuley, R. G. Compton, Chemical analysis in saliva and the search for salivary biomarkers–A tutorial review. *Analyst* **143**, 81–99 (2017).

52. Z.-L. Tan, A.-M. Bao, M. Tao, Y.-J. Liu, J.-N. Zhou, Circadian rhythm of salivary serotonin in patients with major depressive disorder. *Neuroendocrinol. Lett.* **28**, 395–400 (2007).

53. S. S. Dickerson, M. E. Kemeny, Acute stressors and cortisol responses: A theoretical integration and synthesis of laboratory research. *Psychol. Bull.* **130**, 355–391 (2004).

54. A. Papadimitriou, K. N. Priftis, Regulation of the hypothalamic-pituitary-adrenal axis. *Neuroimmunomodulation* **16**, 265–271 (2009).

55. C. A. Elverson, M. E. Wilson, Cortisol: Circadian rhythm and response to a stressor. *Newborn Infant Nurs Rev* **5**, 159–169 (2005).

56. M. A. Birkett, The Trier Social Stress Test protocol for inducing psychological stress. *J. Vis. Exp.* **56**, e3238 (2011).

57. U. Knutsson, J. Dahlgren, C. Marcus, S. Rosberg, M. Brönnegård, P. Stierna, K. Albertsson-Wikland, Circadian cortisol rhythms in healthy boys and girls: Relationship with age, growth, body composition, and pubertal development. *J. Clin. Endocrinol. Metab.* **82**, 536–540 (1997).

58. J. M. Smyth, M. C. Ockenfels, A. A. Gorin, D. Catley, L. S. Porter, C. Kirschbaum, D. H. Hellhammer, A. A. Stone, Individual differences in the diurnal cycle of cortisol. *Psychoneuroendocrinology* **22**, 89–105 (1997).

59. E. Kaufman, I. B. Lamster, The diagnostic applications of saliva—A review. *Crit. Rev. Oral Biol. Med.* **13**, 197–212 (2002).

60. J. M. Yoshizawa, C. A. Schafer, J. J. Schafer, J. J. Farrell, B. J. Paster, D. T. Wong, Salivary biomarkers: Toward future clinical and diagnostic utilities. *Clin. Microbiol. Rev.* **26**, 781–791 (2013).

61. H. Pontzer, J. H. Holloway, D. A. Raichlen, D. E. Lieberman, Control and function of arm swing in human walking and running. *J. Exp. Biol.* **212**, 523–534 (2009).

62. Q. Liu, N. Aroonyadet, Y. Song, X. Wang, X. Cao, Y. Liu, S. Cong, F. Wu, M. E. Thompson, C. Zhou, Highly sensitive and quick detection of acute myocardial infarction biomarkers using In_2O_3 nanoribbon biosensors fabricated using shadow masks. *ACS Nano* **10**, 10117–10125 (2016).

63. N. Aroonyadet, X. Wang, Y. Song, H. Chen, R. J. Cote, M. E. Thompson, R. H. Datar, C. Zhou, Highly scalable, uniform, and sensitive biosensors based on top-down indium oxide

nanoribbons and electronic enzyme-linked immunosorbent assay. *Nano Lett.* **15**, 1943–1951 (2015).

64. S. Emaminejad, W. Gao, E. Wu, Z. A. Davies, H. Y. Y. Nyein, S. Challa, S. P. Ryan, H. M. Fahad, K. Chen, Z. Shahpar, S. Talebi, C. Milla, A. Javey, R. W. Davis, Autonomous sweat extraction and analysis applied to cystic fibrosis and glucose monitoring using a fully integrated wearable platform. *Proc. Natl. Acad. Sci. U.S.A.* **114**, 4625–4630 (2017).

65. H. Lin, J. Tan, J. Zhu, S. Lin, Y. Zhao, W. Yu, H. Hojaiji, B. Wang, S. Yang, X. Cheng, Z. Wang, E. Tang, C. Yeung, S. Emaminejad, A programmable epidermal microfluidic valving system for wearable biofluid management and contextual biomarker analysis. *Nat. Commun.* **11**, 4405 (2020).

66. H. Hojaiji, Y. Zhao, M. C. Gong, M. Mallajosyula, J. Tan, H. Lin, A. M. Hojaiji, S. Lin, C. Milla, A. M. Madni, S. Emaminejad, An autonomous wearable system for diurnal sweat biomarker data acquisition. *Lab Chip* **20**, 4582–4591 (2020).

67. B. R. Baker, R. Y. Lai, M. S. Wood, E. H. Doctor, A. J. Heeger, K. W. Plaxco, An electronic, aptamer-based small-molecule sensor for the rapid, label-free detection of cocaine in adulterated samples and biological fluids. *J. Am. Chem. Soc.* **128**, 3138–3139 (2006).

68. Y. Xiao, R. Y. Lai, K. W. Plaxco, Preparation of electrode-immobilized, redox-modified oligonucleotides for electrochemical DNA and aptamer-based sensing. *Nat. Protoc.* **2**, 2875–2880 (2007).

69. S. Lin, W. Yu, B. Wang, Y. Zhao, K. En, J. Zhu, X. Cheng, C. Zhou, H. Lin, Z. Wang, H. Hojaiji, C. Yeung, C. Milla, R. W. Davis, S. Emaminejad, Noninvasive wearable electroactive pharmaceutical monitoring for personalized therapeutics. *Proc. Natl. Acad. Sci. U.S.A.* **117**, 19017–19025 (2020).

70. P. Blanck, S. Perleth, T. Heidenreich, P. Kröger, B. Ditzen, H. Bents, J. Mander, Effects of mindfulness exercises as stand-alone intervention on symptoms of anxiety and depression: Systematic review and meta-analysis. *Behav. Res. Ther.* **102**, 25–35 (2018).

71. A. Maijala, H. Kinnunen, H. Koskimäki, T. Jämsä, M. Kangas, Nocturnal finger skin temperature in menstrual cycle tracking: Ambulatory pilot study using a wearable Oura ring. *BMC Womens Health* **19**, 150 (2019).

72. J. Hu, C. J. Easley, A simple and rapid approach for measurement of dissociation constants of DNA aptamers against proteins and small molecules via automated microchip electrophoresis. *Analyst* **136**, 3461–3468 (2011).

73. A. Renaud de la Faverie, A. Guedin, A. Bedrat, L. A. Yatsunyk, J.-L. Mergny, Thioflavin T as a fluorescence light-up probe for G4 formation. *Nucleic Acids Res.* **42**, e65 (2014).

74. M. S. Salahudeen, P. S. Nishtala, An overview of pharmacodynamic modelling, ligand-binding approach and its application in clinical practice. *Saudi Pharm. J.* **25**, 165–175 (2017).

75. E. C. Hulme, M. A. Trevethick, Ligand binding assays at equilibrium: Validation and interpretation. *Br. J. Pharmacol.* **161**, 1219–1237 (2010).

76. M. M. Van Eck, N. A. Nicolson, Perceived stress and salivary cortisol in daily life. *Ann. Behav. Med.* **16**, 221–227 (1994).

77. Y. S. Shin, J. N. Liu, J.-H. Kim, Y.-H. Nam, G. S. Choi, H.-S. Park; Premier Researchers Aiming New Era in Asthma and Allergic Diseases (PRANA) Study Group, The impact of asthma control on salivary cortisol level in adult asthmatics. *Allergy Asthma Immunol. Res.* **6**, 463–466 (2014).

78. S. L. King, K. M. Hegadoren, Stress hormones: How do they measure up? *Biol. Res. Nurs.* **4**, 92–103 (2002).

79. F. Elio, G. Antonelli, A. Benetazzo, M. Prearo, R. Gatti, Human saliva cortisone and cortisol simultaneous analysis using reverse phase HPLC technique. *Clin. Chim. Acta* **405**, 60–65 (2009).

80. M. Moriarty, A. Lee, B. O'Connell, A. Kelleher, H. Keeley, A. Furey, Development of an LC-MS/MS method for the analysis of serotonin and related compounds in urine and the identification of a potential biomarker for attention deficit hyperactivity/hyperkinetic disorder. *Anal. Bioanal. Chem.* **401**, 2481–2493 (2011).

81. R. Zhang, Y. Jia, A disposable printed liquid gate graphene field effect transistor for a salivary cortisol test. *ACS Sens.* **6**, 3024–3031 (2021).

82. M. Ku, J. Kim, J.-E. Won, W. Kang, Y.-G. Park, J. Park, J.-H. Lee, J. Cheon, H. H. Lee, J.-U. Park, Smart, soft contact lens for wireless immunosensing of cortisol. *Sci. Adv.* **6**, eabb2891 (2020).

83. N. K. Singh, S. Chung, M. Sveiven, D. A. Hall, Cortisol detection in undiluted human serum using a sensitive electrochemical structure-switching aptamer over an antifouling nanocomposite layer. *ACS Omega* **6**, 27888–27897 (2021).
